# Characterizing lineage-specific evolution and the processes driving genomic diversification in chordates

**DOI:** 10.1186/s12862-020-1585-y

**Published:** 2020-02-11

**Authors:** David E. Northover, Stephen D. Shank, David A. Liberles

**Affiliations:** 1 Department of Biology and Center for Computational Genetics and Genomics, Temple University, Philadelphia, PA 19122 USA; 2 Department of Molecular Biology, University of Wyoming, Laramie, WY 82071 USA

**Keywords:** Comparative genomics, Molecular evolution, Gene duplication, Pathway evolution, Protein structure

## Abstract

**Background:**

Understanding the origins of genome content has long been a goal of molecular evolution and comparative genomics. By examining genome evolution through the guise of lineage-specific evolution, it is possible to make inferences about the evolutionary events that have given rise to species-specific diversification. Here we characterize the evolutionary trends found in chordate species using The Adaptive Evolution Database (TAED). TAED is a database of phylogenetically indexed gene families designed to detect episodes of directional or diversifying selection across chordates. Gene families within the database have been assessed for lineage-specific estimates of dN/dS and have been reconciled to the chordate species to identify retained duplicates. Gene families have also been mapped to the functional pathways and amino acid changes which occurred on high dN/dS lineages have been mapped to protein structures.

**Results:**

An analysis of this exhaustive database has enabled a characterization of the processes of lineage-specific diversification in chordates. A pathway level enrichment analysis of TAED determined that pathways most commonly found to have elevated rates of evolution included those involved in metabolism, immunity, and cell signaling. An analysis of protein fold presence on proteins, after normalizing for frequency in the database, found common folds such as Rossmann folds, Jelly Roll folds, and TIM barrels were overrepresented on proteins most likely to undergo directional selection. A set of gene families which experience increased numbers of duplications within short evolutionary times are associated with pathways involved in metabolism, olfactory reception, and signaling. An analysis of protein secondary structure indicated more relaxed constraint in β-sheets and stronger constraint on alpha Helices, amidst a general preference for substitutions at exposed sites. Lastly a detailed analysis of the ornithine decarboxylase gene family, a key enzyme in the pathway for polyamine synthesis, revealed lineage-specific evolution along the lineage leading to Cetacea through rapid sequence evolution in a duplicate gene with amino acid substitutions causing active site rearrangement.

**Conclusion:**

Episodes of lineage-specific evolution are frequent throughout chordate species. Both duplication and directional selection have played large roles in the evolution of the phylum. TAED is a powerful tool for facilitating this understanding of lineage-specific evolution.

## Background

As closely related species diverge after a speciation event, their genomes begin to accumulate changes that lead to molecular and phenotypic divergence. Speciation itself is a complex process in chordates that results from the gradual cessation of gene flow. As the isolated populations become separate species, mutations of different magnitudes affect the protein coding repertoire of the two diverging genomes. These changes include synonymous changes that only affect the nucleotide sites, nonsynonymous changes that affect the amino acid sites, and gene duplication and loss events, among other types of changes. A resource comparing chordate genomes in a phylogenetic context, The Adaptive Evolution Database (TAED) has recently been re-generated [33] extending previous versions that were released [46, 66].

The latest version of TAED contains gene families constructed systematically across chordate species as described in Hermansen et al. [33]. Gene families have been filtered for alignment quality and to prevent synonymous site saturation, with the oldest nodes in each rooted gene tree reflecting a speciation event of maximum age being the root of the chordate divergence. All pairwise alignments within each multiple sequence alignment had no more than 10% gaps and were at least 80% identical in non-gapped positions. This then created a trade-off between gene family ages (many had root nodes younger than the last common ancestor of chordates) and alignment quality, although homologous gene family relationships can still be identified through TAED. Gene families have been reconciled to the NCBI taxonomy [67] as a reference species tree and events of positive directional and diversifying selection detected using nonsynonymous to synonymous nucleotide substitution rate ratios in the branches model averaged across sites [83]. Gene families have also been used to identify duplication events using the SoftParsMap parsimony-based gene tree-species tree reconciliation software [9].

In addition to previous iterations of TAED, other studies have also sought to characterize the lineage-specific evolution of chordate genomes. This includes the generation of the Selectome Database [51] from Ensembl [2] data. Selectome extends gene family data automatically generated through the Ensembl pipeline which contains sequences from 68 different genomes. Gene families in Selectome are passed through stringent quality control steps following which tests of selection using branch-site models are implemented against tree topologies from Ensembl. While both Ensembl and Selectome examine evolution in a lineage-specific context, the method by which selection is detected varies, with Ensembl using pairwise analyses to calculate the normalized rate of nonsynonymous to synonymous substitutions (dN/dS) and Selectome using branch-site models of selection based on phylogenetic trees. Pairwise estimates of dN/dS do not account for phylogenetic information which limits the ability to understand evolution in a lineage-specific context, and prohibits detection of directional or diversifying selection on internal lineages. Branch-site models and branch models differ in their sensitivity (power) and selectivity (detection of false positives) [5, 25]. dS saturation is a potential problem for these approaches, with accuracy declining at dS ~ 3 [6].

Gene duplication is another important process to consider when assessing lineage-specific processes of evolution. As genes duplicate, they may undergo different evolutionary pressures and be either neofunctionalized, subfunctionalized, or pseudogenized [42]. In the classical model [55], duplicate gene copies can acquire mutations that lose (pseudogenize), change or gain (neofunctionalize) function mutations when the other copy retains the original function. Neofunctionalization, which can also occur to a gene subsequent to initial subfunctionalization, emerges as the dominant driver of evolution in duplicated genes in this model [35, 65]. As such it is one driver of lineage-specific differences in genome content. Subfunctionalization, the subdividing of functions from an ancestral state, can also lead to lineage-specific functional divergence of genes, without the gain of new functions in the genome as a whole. Without gene duplication as a source of genetic content unconstrained by negative selection, evolution tends to act in a conservative fashion [55].

TAED also presents a picture of lineage-specific evolution using pathway and structural information in addition to selection on individual protein encoding genes and gene duplication. Pathway level analyses of proteins may lead to understanding how proteins evolve in the context of a cell or organism, since proteins typically interact together in a pathway or network to achieve biological functions (phenotypes). Simulations have suggested that rate limiting steps are not evolutionarily stable over longer evolutionary periods [56, 57] and proteins currently involved in rate limiting steps may not remain so over long evolutionary periods. This suggests patterns that might be expected for gene-specific selective pressures in a pathway and how they relate to phenotypic evolution.

Two models for the evolution of pathways have been presented, the retrograde evolution model [34], proposing evolution to build a pathway backwards from the selected final product based upon affinity for related transition states at neighboring positions of a pathway and the patchwork model [38] suggesting that gene duplication retains catalytic mechanisms on widely distributed substrates that are dispersed throughout the network of pathways. A driver of mutational opportunity in both models is gene duplication. Analysis of protein function can identify which model is best associated with the evolution of a given pathway, with evidence suggesting that the patchwork model is more common [48]. TAED compiles duplication and selection data compiled for pathways in a lineage-specific manner that can be viewed in this light.

Understanding the structural context of substitutions within a protein may elucidate the role of individual amino acid changes in potential functional shifts under positive selection, differentiating them from compensatory or stabilizing substitutions within the protein. Modeling the effects of amino acid substitutions can demonstrate changes in structure, dynamics, allosteric regulation, and ligand binding that can be used to identify functional shifts ([19]; see also [16]). Such modeling is limited however as the process is difficult and computationally intensive, with identification of fitness effects based upon biophysical models inexact. Measurements and models based on experimental work can also contribute to our understanding [14].

The structural context of mutations also impacts the substitution rate via negative selection. Requirements for folding stability drive lower substitutions in the protein core, while binding requirements on the ligand interface slow mutation as compared to the protein surface [28]. These constraints extend to functional requirements to avoid certain alternate states, including both selection against alternate folding states and substrates that result in deleterious interactions [47]. As protein structure diverges less observably than protein sequence over equivalent units of evolutionary time [36], similar structural constraints can be assumed to be approximately equivalently applicable to sequences diverged over relatively short evolutionary times.

Understanding how genes evolve and the processes by which they lead to novel adaptations in species is fundamental to understanding the genotype-phenotype map. Here we present some new characterizations of lineage-specific evolution utilizing the TAED database; we examine specific hypotheses across lineages, as well as characterizing processes at the levels of gene duplication, pathway evolution, and of protein structure.

## Results

The Adaptive Evolution Database (TAED) contains ~ 3.2 million sequences from 3214 different chordate species. The database contains 143,806 individual genes families which are mapped to the chordate species tree. Twenty-three thousand nine hundred seventy gene families contained one or more branches with dN/dS > 1, indicating positive or directional selection acting on these lineages. When the dN/dS rates are high after controlling for dS saturation, the lineages are candidates for having undergone functional shifts. It is expected that the larger the dN/dS value for a given branch, the stronger the putative selective forces were to cause functional changes to the ancestral protein [73]. A list of the lineages with the largest dN/dS values where dS > 0.01 was generated, as these proteins constituted potential strong candidates for having undergone positive selection (Table 1). Of the top 30 lineages with the largest dN/dS values, values were found to range from 88.78 to 26.57. The families that these proteins come from are putatively involved in multiple different biological processes, many of which do not map to a KEGG pathway. Interestingly strong selection was found to have occurred on the branch leading from Boreoeutherian mammals in 9 of the top 30 instances of high dN/dS. This lineage constitutes species before the split of Laurasiatheria and Euarchontoglires, following the divergence of mammals. Additionally, strong selection was seen repeatedly on the lineage leading from Laurasiatheria which is the superorder containing cetaceans, carnivores, chiropterans, and ruminants. Functional shifts in these proteins may be responsible for some of the physiological and habitat differences between these groups and shared ancestors with carnivores and primates. Strong selection was seen to occur on the lineage leading from Neognathae which comprises most avian species. Pathways under selection along this lineage may indicate some of the functional differences between flightless birds which comprise the sister order Palaeognathae and other avians. KEGG pathway mappings for the top 30 lineages with high dN/dS showed that selection may have acted on several different pathway types including metabolic pathway interactions, receptor signaling pathways, and immune response pathways. Selection can act directly on many different levels within an organism. It can occur at the DNA level, the protein level, the pathway level, and the phenotypic level. Understanding pathway evolution may ultimately be a better way to assess selection than current codon based methods [32].

**Table 1:** TAED gene family lineages with the largest dN/dS values where dS > 0.001

TAED Gene Families with high dN/dS				
Family	dN/dS Value	Mapped Location on Chordate Species Tree (Start to End)	Family description	KEGG Pathways
151,766	88.7785	Boreoeutheria to Laurasiatheria	splicing factor arginine/serine-rich 4/5/6	Herpes simplex infection; Spliceosome
30	76.4909	Cercopithecidae to Cercopithecidae	transmembrane protein 91 isoform X1	
60,787	63.0029	Euarchontoglires to Simiiformes	fucose-1-phosphate guanylyltransferase	Metabolic pathways; Fructose and mannose metabolism; Amino sugar and nucleotide sugar metabolism
23,133	61.9262	Aves to Aves	galanin receptor 1	Neuroactive ligand-receptor interaction
296,346	55.184	Eutheria to Delphinidae	LOW QUALITY PROTEIN: probable N-acetyltransferase 16	
21,900	45.0176	Boreoeutheria to Boreoeutheria	X-linked interleukin-1 receptor accessory protein-like 2	
52,181	44.575	Boreoeutheria to Laurasiatheria	unnamed protein product	
8186	44.3077	Sauria to *Anolis carolinensis*	protein Simiate	
9378	40.3796	Neognathae to Neognathae	palmitoyltransferase ZDHHC17 isoform X4	
22,600	39.9557	Boreoeutheria to Laurasiatheria	potassium voltage-gated channel subfamily G member 3 isoform X1	
4144	38.9415	Hystricognathi to Hystricognathi	pygopus homolog 1	
14,875	38.1267	Laurasiatheria to Laurasiatheria	kinesin-like protein KIF16B	
12,213	38.0258	Camelidae to Camelus	LOW QUALITY PROTEIN: dnaJ homolog subfamily B member 3	
12,593	37.1258	Boreoeutheria to Boreoeutheria	tRNA (guanine(10)-N2)-methyltransferase homolog	
20,708	36.782	Amniota to Amniota	heparan sulfate 2-O-sulfotransferase HS2ST1	Glycosaminoglycan biosynthesis - heparan sulfate / heparin
32,532	36.0215	Boreoeutheria to *Carlito syrichta*	interleukin 8	AGE-RAGE signaling pathway in diabetic complications; NOD-like receptor signaling pathway;Influenza A; Phospholipase D signaling pathway; Chemokine signaling pathway;Hepatitis B; Toll-like receptor signaling pathway; Legionellosis;RIG-I-like receptor signaling pathway; Rheumatoid arthritis; Malaria; NF-kappa B signaling pathway; Shigellosis;Hepatitis C;N on-alcoholic fatty liver disease (NAFLD); Pathways in cancer; Epithelial cell signaling in *Helicobacter pylori* infection; Amoebiasis; Bladder cancer; IL-17 signaling pathway; Chagas disease (*American trypanosomiasis*); Pertussis; Transcriptional misregulation in cancer; Salmonella infection; Cytokine-cytokine receptor interaction
4273	35.4369	Laurasiatheria to Laurasiatheria	myelin protein zero	Cell adhesion molecules (CAMs)
21,944	35.0114	Boreoeutheria to Boreoeutheria	adrenergic receptor alpha-2C	cGMP-PKG signaling pathway; Neuroactive ligand-receptor interaction
14,303	34.6434	Neognathae to Neognathae	ATP-dependent DNA helicase PIF1 partial	
14,588	34.0198	Neognathae to Neognathae	organic solute transporter subunit alpha-like partial	
12,299	34.0037	Neognathae to Neognathae	phosphatidylinositol glycan class H	Glycosylphosphatidylinositol (GPI)-anchor biosynthesis; Metabolic pathways
8762	33.6089	Myotis to Myotis	doublesex- and mab-3-related transcription factor 2 isoform X1	
55,196	31.2663	Murinae to *Mus musculus*	Isx protein	
14,119	31.0551	Passeriformes to Passeriformes	cardiolipin synthase CMP-forming	Glycerophospholipid metabolism; Metabolic pathways
39	30.43	Boreoeutheria to Boreoeutheria	large subunit ribosomal protein L4e	Ribosome
23,596	30.0168	Neognathae to Neognathae	diacylglycerol cholinephosphotransferase	Phosphonate and phosphinate metabolism; Glycerophospholipid metabolism; Metabolic pathways; Choline metabolism in cancer; Ether lipid metabolism
10,501	29.8433	*Homo sapiens* to *Homo sapiens*	smoothelin isoform X5	
157,103	27.6427	Neognathae to *Haliaeetus albicilla*	nucleolar protein 7 partial	
3717	27.4352	Boreoeutheria to Boreoeutheria	glypican 4	Wnt signaling pathway
9725	26.5684	Eutheria to Boreoeutheria	cilia- and flagella-associated protein 221-like partial	

### Enrichment analysis

To gain a better understanding of pathways within TAED that are more common targets of directional selection, a test to determine which pathways were over or under represented for instances of putative positive selection was undertaken. Table 2 shows the list of the top 25 enriched KEGG pathways within TAED for directional selection. From the top 25 pathways that are over-represented in the database, 8 of the pathways are involved in metabolic reactions (the pathway labeled “Metabolic pathways” contains proteins from all metabolic pathways, and therefore is not a unique pathway). Metabolism, or the process of constructing useful cellular molecules, is essential for life. Given the vast array of different physiological and environmental conditions that exist within chordate species, it is plausible that developing different metabolic strategies is a primary way for organisms to cope with their surroundings. As such, seeing that these pathways are often targets for directional selection is not surprising. Furthermore, it is evident from the list that pathways involved in immune response and cellular health have also been directly impacted by selection. Over-represented pathways involved in immune response included: Herpes simplex infection, Influenza A, Toxoplasmosis, and Th17 cell differentiation. It has been documented in the literature that selection against pathogens is a constant arms race that requires novel adaptations to overcome the constant pressures of pathogenic infection [15, 44, 78]; that these pathways should be over-represented for putative positive selection is not surprising. Additionally, pathways which alleviate physiological stress also appear to be over-represented for directional selection as seen in the pathways: fluid shear stress and atherosclerosis, non-alcoholic fatty liver disease, and chemical carcinogenesis. Cellular components were also found to be under selective pressure to evolve as seen in the pathways, protein processing in endoplasmic reticulum, RNA transport, lysosome, and peroxisome. Lastly, many lineages were found to have evolved under directional selection relating to olfactory transduction. Olfactory genes are the most duplicated genes within the human genome and are known to be largely expanded in other chordate species [54]. Olfactory sense is a primary means of communication, predation, and foraging for many species and thus is unsurprising that many lineages relating to this pathway have instances of dN/dS > 1.

**Table 2:** Pathways present in lineages under positive selection

Over-Represented KEGG Pathways TAED					
KEGG Pathway	Mapped Lineages Under Positive Selection	Lineages Under Positive Selection Mapped	Uncorrected *P*-value	FDR *P*-value	Bonferroni *P*-value
Metabolic pathways	7.63%	5.73%	< 0.0001	< 0.0001	< 0.0001
Olfactory transduction	12.25%	2.67%	< 0.0001	< 0.0001	< 0.0001
Biosynthesis of secondary metabolites	7.85%	1.90%	< 0.0001	< 0.0001	< 0.0001
Biosynthesis of antibiotics	7.96%	1.11%	< 0.0001	< 0.0001	< 0.0001
Neuroactive ligand-receptor interaction	6.45%	1.05%	< 0.0001	< 0.0001	< 0.0001
Microbial metabolism in diverse environments	7.86%	0.85%	< 0.0001	< 0.0001	< 0.0001
Protein processing in endoplasmic reticulum	6.97%	0.72%	< 0.0001	< 0.0001	< 0.0001
Purine metabolism	6.63%	0.69%	< 0.0001	< 0.0001	< 0.0001
Herpes simplex infection	6.18%	0.68%	0.0047	0.0128	**1.0000**
Carbon metabolism	8.63%	0.62%	< 0.0001	< 0.0001	< 0.0001
RNA transport	6.45%	0.59%	< 0.0001	< 0.0001	0.0056
Influenza A	6.74%	0.59%	< 0.0001	< 0.0001	< 0.0001
Fluid shear stress and atherosclerosis	6.21%	0.54%	0.0061	0.016	**1.0000**
Lysosome	6.72%	0.52%	< 0.0001	< 0.0001	< 0.0001
Glycerophospholipid metabolism	7.61%	0.49%	< 0.0001	< 0.0001	< 0.0001
Non-alcoholic fatty liver disease (NAFLD)	6.19%	0.47%	0.0128	0.0325	**1.0000**
Pancreatic secretion	8.86%	0.43%	< 0.0001	< 0.0001	< 0.0001
Peroxisome	7.37%	0.42%	< 0.0001	< 0.0001	< 0.0001
Toxoplasmosis	6.50%	0.41%	0.0001	0.0003	0.0341
Phosphatidylinositol signaling system	6.70%	0.41%	< 0.0001	< 0.0001	0.0004
Glycerolipid metabolism	10.21%	0.39%	< 0.0001	< 0.0001	< 0.0001
Drug metabolism - cytochrome P450	12.03%	0.39%	< 0.0001	< 0.0001	< 0.0001
Th17 cell differentiation	6.38%	0.38%	0.0013	0.0039	**0.4944**
Valine_ leucine and isoleucine degradation	11.00%	0.38%	< 0.0001	< 0.0001	< 0.0001
Chemical carcinogenesis	9.32%	0.38%	< 0.001	< 0.0001	< 0.0001

The top 25 over-represented KEGG pathways with the highest % of lineages under positive selection mapping to a pathway. All pathways were significant at the 0.05 level after correction with the false discovery rate (FDR). Bold numbers indicate not significant at the 0.05 level. Lineages with dN/dS > 1 considered under positive selection

Of the pathways found within TAED to be under-represented for functional shifts, surprisingly phototransduction was found to be included within the top 25 (Table 3). The ability to visually see pigments is important in both sexual selection and predation. In birds [12, 84], fish ([72, 74, 79];) and cetaceans [24] instances of positive selection have been discovered relating to selection on opsin and rhodopsin genes. Therefore, it is surprising that selection on this KEGG pathway would be under-represented within TAED. However, KEGG pathways for zeatin biosynthesis, penicillin and cephalosporin biosynthesis, bacterial secretion systems, and MAPK signaling pathway – plant, should be underrepresented in the database as these pathways are primarily involved in either plant or microbial systems and do not constitute meaningful pathways in chordates although orthologous proteins to some of the components to these pathways do exist in chordates, but may have different functions. RNA polymerase is a highly-conserved protein found throughout all domains of life, and therefore is unsurprising that the pathway for RNA polymerase would be under-represented for functional shifts within chordate species.

**Table 3:** Pathways absent in lineages under positive selection

Under-Represented KEGG pathways TAED					
KEGG Pathway	Mapped Lineages Under Positive Selection	Lineages Under Positive Selection Mapped	Uncorrected *P*-value	FDR *P*-value	Bonferroni *P*-value
Zeatin biosynthesis	0.53%	< 0.01%	0.0001	0.0006	**0.0562**
D-Arginine and D-ornithine metabolism	1.12%	< 0.01%	0.0016	0.0056	**0.5896**
Penicillin and cephalosporin biosynthesis	1.12%	< 0.01%	0.0016	0.0056	**0.5896**
Indole alkaloid biosynthesis	0.89%	< 0.01%	< 0.0001	< 0.0001	0.0011
Bacterial secretion system	1.40%	< 0.01%	0.0013	0.0047	**0.4768**
Toluene degradation	2.53%	0.01%	0.0138	0.0422	**1.0000**
Fluorobenzoate degradation	2.53%	0.01%	0.0138	0.0422	**1.0000**
Chlorocyclohexane and chlorobenzene degradation	2.53%	0.01%	0.0138	0.0422	**1.0000**
Styrene degradation	1.37%	0.01%	< 0.0001	< 0.0001	< 0.0001
Tropane_ piperidine and pyridine alkaloid biosynthesis	3.25%	0.02%	0.0014	0.0052	**0.5283**
Cyanoamino acid metabolism	4.07%	0.06%	0.0023	0.0080	**0.8550**
Maturity onset diabetes of the young	4.11%	0.08%	0.0004	0.0014	**0.1353**
Phototransduction - fly	1.64%	0.09%	< 0.0001	< 0.0001	< 0.0001
MAPK signaling pathway - plant	4.42%	0.11%	0.0014	0.0052	**0.5399**
Glycosaminoglycan biosynthesis - keratan sulfate	3.51%	0.11%	< 0.0001	< 0.0001	< 0.0001
Glycosaminoglycan biosynthesis - heparan sulfate / heparin	4.06%	0.11%	< 0.0001	0.0001	0.0061
Thyroid cancer	2.78%	0.12%	< 0.0001	< 0.0001	< 0.0001
Mannose type O-glycan biosynthesis	4.56%	0.15%	0.0010	0.0037	**0.3723**
RNA polymerase	2.96%	0.15%	< 0.0001	< 0.0001	< 0.0001
Glycosaminoglycan biosynthesis - chondroitin sulfate / dermatan sulfate	4.95%	0.16%	0.0171	0.0506	**1.0000**
Phototransduction	3.79%	0.17%	< 0.0001	< 0.0001	< 0.0001
Nicotine addiction	4.07%	0.21%	< 0.0001	< 0.0001	< 0.0001
Collecting duct acid secretion	5.06%	0.22%	0.0158	0.0472	**1.0000**
Pathogenic *Escherichia coli* infection	3.61%	0.23%	< 0.0001	< 0.0001	< 0.0001
Hedgehog signaling pathway - fly	3.88%	0.23%	< 0.0001	< 0.0001	< 0.0001

The KEGG pathways with the lowest % of lineages under positive selection mapping to a pathway. All pathways were significant at the 0.05 level after correction with the false discovery rate (FDR). Bold numbers indicate not significant at the 0.05 level. Lineages with dN/dS > 1 considered under positive selection

Another interesting question which was generated from structural elements contained in TAED was if some functional protein domains are more likely to experience elevated rates of evolution compared to others. To determine if this is true a systematic search was performed to determine what functional domain topologies are enriched within lineages in TAED that have signals for functional change (Table 4). Functional domains were annotated from the CATH database which assigns each domain a CATH classification. Annotations for this analysis looked at the topology level as it contains a wide array of functional domain annotations. The most over-represented domain/fold within TAED was the Rossmann fold which constituted approximately a quarter of all lineages in TAED with dN/dS > 1 that could map to a domain (the analysis did normalize for abundance in the database). The Rossmann fold is a common fold comprised of a b-a-b-a-b (b – beta sheet, a – alpha helix) subunit motif and is commonly found within nucleotide-binding proteins [63]. Proteins that include this fold type include kinases, guanine nucleotide binding proteins (G proteins), proteins that bind cyclic adenosine monophosphate (cAMP), and NAD(P)-binding proteins [31]. These proteins are abundant within a cell and therefore proteins in which these domains reside are likely candidates for directional selection. However due to the nature and importance of nucleotide binding, it is unlikely that the Rossmann fold is under selection, but other domains within the same protein are as this domain is likely under strong negative constraint unless there are selective pressures on binding affinity or specificity. More structural analyses of the lineages under selection that contain the Rossmann fold would be warranted to examine this in more detail. The second most over represented domain topology was the Jelly Rolls fold which a subset of the beta-barrels superfamily. This fold type is composed of 8 beta-sheets which fold into a roll shape [1]. These folds are commonly found in viral capsid proteins [64]. It is possible that since these folds are commonly found in viral proteins that they evolve quickly and are prone to high mutation rates. This would suggest that protein families which contain this domain would be over-represented. The third most over-represented domain topology was TIM barrel folds. These are very common folds found with proteins that share alpha-beta structures. The TIM barrel folds are known to be highly promiscuous in sequence with many different sequences able to generate the TIM barrel fold. Therefore, there is biophysical flexibility for amino acids within these domains to be substituted while still maintaining the same domain structure [82]. These folds are in some cases known over longer evolutionary periods as folds that are structurally adaptable and evolve under relaxed selective constraint [17, 27, 45], consistent with their observation here in divergence among closely related species.

**Table 4:** Domains present in lineages under positive selection

Over-Represented CATH Domain Topologies in TAED					
CATH domain topology	Mapped Lineages Under Positive Selection	Lineages Under Positive Selection Mapped	Uncorrected *P*-value	FDR *P*-value	Bonferroni *P*-value
Rossmann fold	7.35%	25.55%	< 0.0001	< 0.0001	< 0.0001
Jelly Rolls	7.30%	4.43%	< 0.0001	< 0.0001	0.0013
Phosphorylase Kinase domain 1	9.51%	3.87%	< 0.0001	< 0.0001	< 0.0001
TIM Barrel	7.52%	3.59%	< 0.0001	< 0.0001	< 0.0001
Thrombin subunit H	11.13%	2.90%	< 0.0001	< 0.0001	< 0.0001
Ubiquitin-like (UB roll)	7.06%	2.50%	0.0143	0.0355	**1.0000**
Glutaredoxin	9.53%	2.39%	< 0.0001	< 0.0001	< 0.0001
Collagenase (Catalytic Domain)	9.70%	2.22%	< 0.0001	< 0.0001	< 0.0001
DNA polymerase domain 1	8.26%	2.12%	< 0.0001	< 0.0001	< 0.0001
OB fold (Dihydrolipoamide Acetyltransferase E2P)	7.32%	1.79%	0.0013	0.0039	**0.8113**
Methane Monooxygenase Hydroxylase Chain G domain 1	8.24%	1.55%	< 0.0001	< 0.0001	< 0.0001
Cytochrome p450	9.11%	1.51%	< 0.0001	< 0.0001	< 0.0001
Helicase Ruva Protein domain 3	9.86%	1.21%	< 0.0001	< 0.0001	< 0.0001
Laminin	8.56%	1.03%	< 0.0001	< 0.0001	< 0.0001
Glutathione S-transferase Yfyf (Class Pi) Chain A domain 2	10.79%	1.01%	< 0.0001	< 0.0001	< 0.0001
Kinesin	8.47%	1.00%	< 0.0001	< 0.0001	< 0.0001
Glycosyltransferase	12.75%	0.92%	< 0.0001	< 0.0001	< 0.0001
FAD/NAD(P)-binding domain	10.68%	0.87%	< 0.0001	< 0.0001	< 0.0001
2-enoyl-CoA Hydratase Chain A domain 1	14.07%	0.80%	< 0.0001	< 0.0001	< 0.0001
Erythroid Transcription Factor GATA-1 Chain A	9.34%	0.72%	< 0.0001	< 0.0001	< 0.0001
Cyclin A domain 1	10.32%	0.71%	< 0.0001	< 0.0001	< 0.0001
Alkaline Phosphatase subunit A	9.96%	0.69%	< 0.0001	< 0.0001	< 0.0001
Butyryl-CoA Dehydrogenase subunit A domain 3	9.13%	0.68%	< 0.0001	< 0.0001	< 0.0001
Carbonic Anhydrase II	14.52%	0.66%	< 0.0001	< 0.0001	< 0.0001

Enrichment analysis of CATH domain topologies in TAED showing CATH domains topologies present in highest % of lineages under positive selection. All pathways were significant at the 0.05 level after correction with the false discovery rate (FDR). Bold numbers indicate not significant at the 0.05 level. Lineages with dN/dS > 1 considered under positive selection

From the list of the top under-represented domain topologies (Table 5), two of the most under-represented domains were derived from the SMAD3 (mothers against decapentaplegic homolog 3) protein (smad3 chain A and Smad anchor for receptor activation chain B). The SMAD3 protein is involved in the signal trafficking of TGF-β which plays an important role in cell growth and death. This protein structure is known to contain two different domains, a DNA-binding domain and a protein-protein interacting domain. These two domains have been shown to be conserved across many species and play an essential role in the function of SMAD proteins [52, 53]. Accordingly, it is expected that these domains would be very limited in the rate at which they evolve and that they would evolve mostly under strong negative selection. Another interesting protein domain that was under-represented within the database was the fold for cAMP-dependent protein kinase. The primary enzyme which contains this domain is protein kinase A (PKA) which is involved in many different cellular pathways and plays a role in cell growth and differentiation, signaling, and migration [21]. As a central hub protein within a protein interaction network, it would be expected that this would be highly negatively constrained [58] and therefore domains that are essential to this protein are also under strong negative selection.

**Table 5:** Domains absent in lineages under positive selection

Under-Represented CATH Domain Topologies in TAED					
CATH domain topology	Mapped Lineages Under Positive Selection	Lineages Under Positive Selection Mapped	Uncorrected *P*-value	FDR *P*-value	Bonferroni *P*-value
Smad3 Chain A	0.08%	< 0.01%	< 0.0001	< 0.0001	< 0.0001
A middle domain of Talin 1	0.24%	< 0.01%	< 0.0001	< 0.0001	< 0.0001
Endonuclease - Pi-scei_ Chain A domain 1	0.27%	< 0.01%	< 0.0001	< 0.0001	< 0.0001
Undecaprenyl pyrophosphate synthetase	0.33%	< 0.01%	< 0.0001	< 0.0001	< 0.0001
Neurophysin II Chain A	0.38%	< 0.01%	< 0.0001	< 0.0001	0.0002
Major Prion Protein	0.43%	< 0.01%	< 0.0001	< 0.0001	0.0010
Archaeosine Trna-guanine Transglycosylase Chain: A domain 4	0.45%	< 0.01%	< 0.0001	< 0.0001	0.0023
Translation Eukaryotic Peptide Chain Release Factor Subunit 1 Chain A	0.52%	< 0.01%	< 0.0001	0.0002	0.0140
PWI domain	0.53%	< 0.01%	< 0.0001	0.0003	0.0206
copper amine oxidase-like fold	0.57%	< 0.01%	0.0001	0.0005	0.0472
ERH-like fold	0.58%	< 0.01%	0.0001	0.0007	0.0608
Smad Anchor For Receptor Activation Chain B	0.61%	< 0.01%	0.0002	0.0010	**0.1011**
protein kinase ck2 holoenzyme chain C domain 1	0.61%	< 0.01%	0.0002	0.0010	**0.1011**
Elongin C Chain C domain 1	0.65%	< 0.01%	0.0003	0.0017	**0.1785**
Conserved hypothetical protein from pyrococcus furiosus pfu- 392,566-001 ParB domain	0.90%	< 0.01%	0.0042	0.0195	**1.0000**
titin filament fold	0.92%	< 0.01%	0.0048	0.0214	**1.0000**
Transcription Regulator spoIIAA	0.98%	< 0.01%	0.0073	0.0311	**1.0000**
cAMP-dependent Protein Kinase Chain A	0.36%	< 0.01%	< 0.0001	< 0.0001	< 0.0001
Inorganic Pyrophosphatase	0.45%	< 0.01%	< 0.0001	< 0.0001	< 0.0001
subunit c (vma5p) of the yeast v-atpase domain 2	0.51%	< 0.01%	< 0.0001	< 0.0001	< 0.0001
Deoxyuridine 5′-Triphosphate Nucleotidohydrolase_ Chain A	0.88%	< 0.01%	< 0.0001	0.0002	0.0135
50s Ribosomal Protein L19e Chain O domain 1	0.96%	< 0.01%	0.0001	0.0005	0.0451
Glutathione Synthetase Chain A domain 3	1.15%	< 0.01%	0.0006	0.0031	**0.3377**
Deoxyhypusine Synthase	1.18%	< 0.01%	0.0007	0.0037	**0.4263**
DNA Excision Repair Uvrb Chain A	1.29%	< 0.01%	0.0017	0.0084	**1.0000**

Enrichment analysis of CATH domain topologies in TAED showing CATH domains topologies present in lowest % of lineages under positive selection. All pathways were significant at the 0.05 level after correction with the false discovery rate (FDR). Bold numbers indicate not significant at the 0.05 level. Lineages with dN/dS > 1 considered under positive selection

### Duplication analysis

**Fig. 1:**
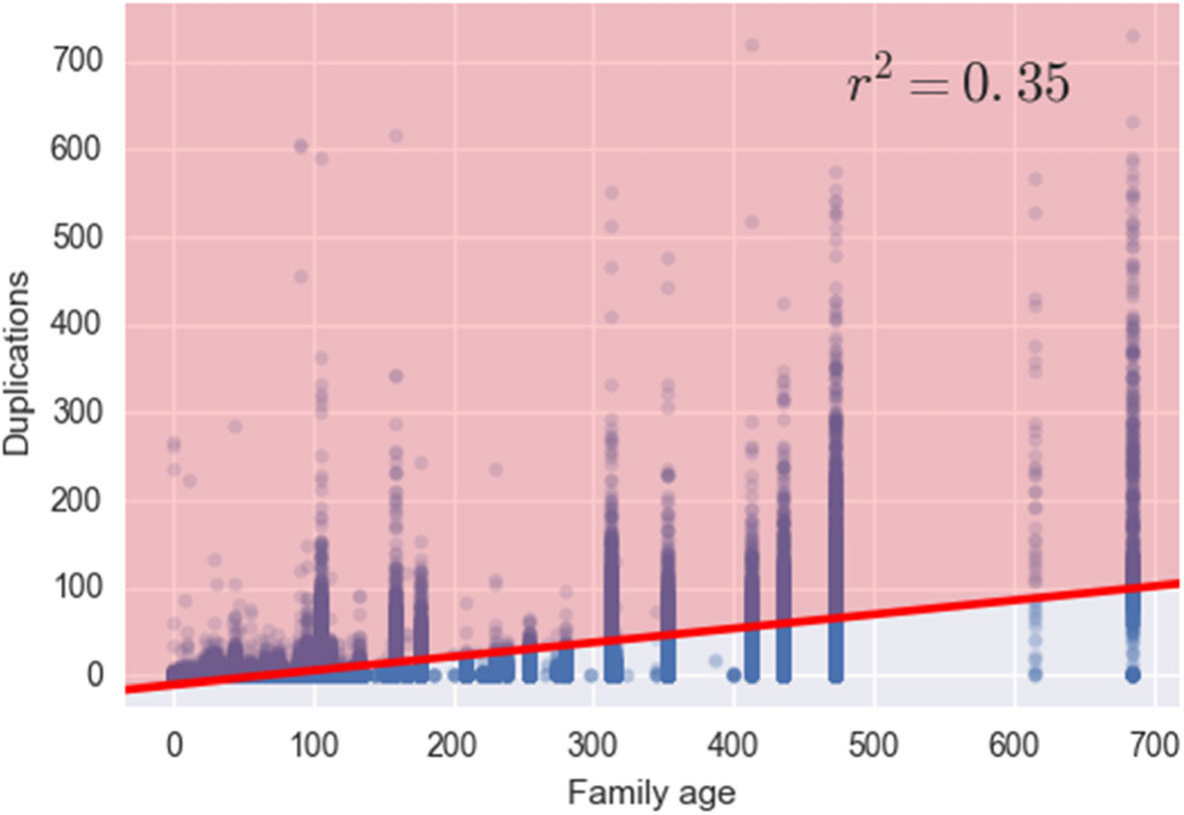
Duplication analysis regression plot using family node ages as a proxy for time – The x-axis is measured in MYA based on the root node for each TAED gene family. The best Pearson’s r coefficient was found when neither axes were log transformed. The upper left half (shaded orange) of the scatterplot was used to determine TAED gene families that were statistically different from the regression line using Cook’s distance

One important element of lineage-specific evolution is the expansion and contraction of genes within the genome. As genes duplicate they may undergo different evolutionary pressures and be either neofunctionalized, subfunctionalized, or pseudogenize [42]. Following the completion of the TAED database, it was interesting to determine if some gene families are more likely to undergo gene duplication events than others and what pathways these genes reside in. Are some pathways more flexible to gene duplication and dosage balance constraints [76] than others? A systematic examination of TAED gene family duplications was performed by scaling the number of duplication events detected within a family by the amount of time over which the family evolved. Three different proxies for time were used in the analysis, the maximum phylogenetic tree length measured in substitutions per site (Additional file 1: Figure S1), the median tree length measured in substitutions per site (Additional file 1: Figure S2), and the relative age of each family found by mapping the root of each gene tree to the chordate species tree (Fig. 1). Each analysis determined that there is a positive correlation between the number of duplications within the family and the amount of time over which the family evolved. Outliers from the regression line identified families that were highly duplicated over a shortened timespan. These families are also those with a high rate of duplication compared to other gene families. Table 6 shows the Cook’s distance calculations for the analysis using family node age as a proxy for time and the corresponding gene families that were calculated to be furthest from the regression line. Cook’s distances for the maximum tree length and median tree length are found in Additional file 1: Tables S1 and S2, respectively. From the families with the largest Cook’s distance the number of times a highly duplicable family mapped to a give KEGG pathways was counted (Table 7). Pathway counts for the maximum tree length and median tree lengths were also calculated (Additional file 1: Tables S3 and S4).

**Table 6:** TAED gene families with many duplications based on family node age from summed branch lengths

Highly duplicable gene families - Family Age			
Cook’s Distance	Family Description	Family Age	Number of Duplications
0.0599	serine/threonine-protein phosphatase 2A 56 kDa regulatory subunit epsilon isoform	684	729
0.0429	receptor-type tyrosine-protein phosphatase F isoform X1	684	632
0.0364	guanine nucleotide-binding protein G(i) subunit alpha-1, partial	684	590
0.0358	peptidyl-prolyl cis-trans isomerase A-like	684	586
0.0338	casein kinase I isoform gamma-2	684	572
0.0328	transcription factor AP-2-alpha isoform X1	684	565
0.0310	protein argonaute-3	684	552
0.0307	serine/threonine-protein phosphatase 2A 55 kDa regulatory subunit B alpha isoform	684	550
0.0303	casein kinase I isoform epsilon	684	547
0.0300	cytoplasmic polyadenylation element-binding protein 4 isoform X1	684	545
0.0283	late histone H2B.L4-like	684	532
0.0266	L-lactate dehydrogenase B chain-like	615	566
0.0264	mitogen-activated protein kinase 11	684	517
0.0262	septin-14	684	516
0.0257	serine/threonine-protein phosphatase 2B catalytic subunit beta isoform isoform X1	684	512
0.0247	alpha-enolase	684	504
0.0247	mitogen-activated protein kinase 10	684	504
0.0240	pre-B-cell leukemia transcription factor 1	684	498
0.0229	heat shock protein HSP 90-beta-like	684	489
0.0229	potassium voltage-gated channel beta subunit	684	489
0.0227	polyadenylate-binding protein 1	615	529
0.0209	protein yippee-like 2	684	471
0.0207	sodium/potassium-transporting ATPase subunit alpha-4 isoform X1	684	469
0.0202	myosin regulatory light polypeptide 9	684	465
0.0200	aldolase B	684	463

**Table 7:** TAED KEGG pathways based on duplication analysis using family node age from summed branch lengths

KEGG Pathway mappings from high duplicable TAED gene families – Family Node Age	
KEGG Pathway	Number of mapping instances from highly duplicable families
Metabolic pathways	558
Olfactory transduction	198
Pathways in cancer	171
PI3K-Akt signaling pathway	130
Endocytosis	130
HTLV-I infection	122
MAPK signaling pathway	121
Proteoglycans in cancer	106
Rap1 signaling pathway	98
Neuroactive ligand-receptor interaction	96
Ras signaling pathway	93
Regulation of actin cytoskeleton	90
Epstein-Barr virus infection	87
Purine metabolism	86
RNA transport	85
Transcriptional misregulation in cancer	83
Protein processing in endoplasmic reticulum	81
Axon guidance	80
mTOR signaling pathway	79
Focal adhesion	79
Viral carcinogenesis	78
Herpes simplex infection	77
cAMP signaling pathway	77
Ribosome	75
Cytokine-cytokine receptor interaction	72

The data shows metabolic pathways and olfactory receptors are consistently the top pathways where duplications occur. Olfactory receptors are known to be the largest expanded gene family [26], aligning our study with the currently known data.

Additionally, the top 25 most highly duplicable gene families included serine/threonine-protein phosphatase 2A 56 kDa regulatory subunit epsilon isoform, abl interactor 1 - partial, aldolase B, guanine nucleotide-binding protein G(i) subunit alpha-1 - partial, and myosin regulatory light polypeptide 9. A further examination of the structural components and pathway components of these families may explain why they are more tolerable to duplication events and the mechanisms that are causing large gene family expansions. Interestingly, many of the most duplicated gene families mapped to KEGG pathways involved in immunity (HTLV-I infection; Herpes simplex infection; Epstein-Barr virus infection; Influenza A) and cancer (Pathways in cancer; Proteoglycans in cancer; Transcriptional misregulation in cancer; Viral carcinogenesis), possibly suggesting that duplication plays a strong role in this arms race.

### Protein structure based analysis

The combination of gene families and information from the Protein Databank allows examination of how selection acts on a protein structural level. Gene families with associated protein structures were collated and aligned to the PDB alongside maximum likelihood ancestral sequences calculated by PAML.

The resulting profile is significantly different than the profile of non-substituted sites in the background on those lineages (Table 8). For both positively and negatively selected lineages, fewer substituted sites are buried relative to all sites on the protein; this is true both looking at all sites, and sites of any specific secondary structure, except for *β-Sheet (p = 0.0361) and β-Bridge* (*p* = 0.0081) sites on positively selected lineages, which was not significant after a multiple testing correction. The result in *β-Bridge sites* may simply be a matter of lower power due to the relatively small number of residues compared to most other secondary structures. *β-Sheet* sites are the most commonly substituted buried site on positive lineages (14.2744% vs 13.1684% for all helices), though *α-Helix* sites, as well as helices in general, are more common amongst all sites (15.9368 and 17.6017% vs 14.5822% for *β-Sheet*).

**Table 8:** Sitewise substitution rates in TAED lineages sorted by selective pressure and structural features

	Positively Selected Lineages (dN/dS > 1)				Negatively Selected Lineages (dN/dS < 0.5)	
	Substituted Sites	*p*	All Sites	Substituted Sites	*p*	All Sites
Helix	30.2826%	**< 0.0001**	34.0597%	35.2377%	**< 0.0001**	36.6327%
Exposed	17.1142%	**0.0002**	16.4580%	20.2511%	**< 0.0001**	17.3397%
Buried	13.1684%	**< 0.0001**	17.6017%	14.9866%	**< 0.0001**	19.2929%
α-Helix	26.1346%	**< 0.0001**	30.1108%	31.1229%	**< 0.0001**	32.7617%
Exposed	14.3764%	0.1659	14.1740%	17.5250%	**< 0.0001**	15.1730%
Buried	11.7582%	**< 0.0001**	15.9368%	13.5979%	**< 0.0001**	17.5887%
3_10_ Helix	3.4956%	0.0098	3.3039%	3.5115%	**< 0.0001**	3.2213%
Exposed	2.3597%	**< 0.0001**	2.0045%	2.4163%	**< 0.0001**	1.8907%
Buried	1.1359%	**0.0004**	1.2994%	1.0952%	**< 0.0001**	1.3306%
π-Helix	0.6524%	0.8047	0.6449%	0.6033%	**< 0.0001**	0.6497%
Exposed	0.3780%	**< 0.0001**	0.2794%	0.3098%	**< 0.0001**	0.2761%
Buried	0.2743%	**0.0005**	0.3655%	0.2935%	**< 0.0001**	0.3736%
β-Sheet	23.2104%	**< 0.0001**	21.7820%	18.2981%	**< 0.0001**	19.8385%
Exposed	8.9360%	**< 0.0001**	7.1998%	7.3255%	**< 0.0001**	6.2661%
Buried	14.2744%	0.0361	14.5822%	10.9726%	**< 0.0001**	13.5724%
β-Bridge	1.1095%	0.7913	1.0984%	0.9888%	**< 0.0001**	1.0382%
Exposed	0.5644%	**0.0004**	0.4641%	0.4876%	**< 0.0001**	0.4194%
Buried	0.5451%	0.0081	0.6343%	0.5012%	**< 0.0001**	0.6188%
Turn	12.0729%	**< 0.0001**	11.0540%	12.3561%	**< 0.0001**	11.0859%
Exposed	9.7554%	**< 0.0001**	8.3283%	9.9588%	**< 0.0001**	8.1517%
Buried	2.3175%	**< 0.0001**	2.7257%	2.3973%	**< 0.0001**	2.9342%
Bend	10.4763%	**< 0.0001**	9.7416%	10.2628%	**< 0.0001**	9.6989%
Exposed	7.9179%	**< 0.0001**	6.8552%	7.8004%	**< 0.0001**	6.6547%
Buried	2.5584%	**< 0.0001**	2.8864%	2.4624%	**< 0.0001**	3.0443%
Coil	22.8482%	**0.0006**	22.2643%	22.8565%	**< 0.0001**	21.7058%
Exposed	15.2151%	**< 0.0001**	13.2976%	15.3522%	**< 0.0001**	12.7858%
Buried	7.6331%	**< 0.0001**	8.9667%	7.5044%	**< 0.0001**	8.9201%
Buried (All Sites)	40.4969%	**< 0.0001**	47.3970%	38.8245%	**< 0.0001**	48.3826%
Exposed (All Sites)	59.5031%	**< 0.0001**	52.6030%	61.1755%	**< 0.0001**	51.6174%

The distribution of substituted sites by secondary structure and solvent accessibility binned by the nautre of selection are shown. Bolded items are significant (*p* < 0.00167 after multiple comparisons correction) based on parametric bootstrapping, *n* = 20000

Negatively selected lineages consistently have an increase in the prevalence of exposed residues across all secondary structures, but this is not universal for positively selected lineages. α-Helix sites are the most frequent in the dataset and show no change in prevalence of exposed sites compared to non-substituted sites under positive selection. *3*_*10*_ *Helix* sites show an overall increase in substitution rates in negatively selected lineages, unlike other helixes but consistent with bends, turns and coil sites. This is likely linked to their lower stability and higher proportion of exposed vs buried sites.

In terms of secondary structure when both exposed and buried regions are considered together, substitutions are more likely to occur across less structured regions (Turns, Bends, and Coil areas) that are more likely to be exposed than buried on both positively and negatively selected lineages, but also *β-Sheet* sites on positively selected lineages and *3*_*10*_ *Helix* sites on negatively selected lineages. The changes in prevalence for each secondary structure is strongly related to the buried/exposed ratio of their own residues (particularly in negatively selected sites), so solvent exposure, while a significant factor, is not the only one. This corresponds with observations seen in other studies ([18] and studies cited therein).

The lack of significant change in *β-Sheet* buried sites on positively selected lineages, suggests that positive selection is freer to act on it than comparable α-Helix sites, which have a considerable drop in frequency amongst substituted (13.1684%) rather than all (17.6017%) sites. The *β-Sheet* site changes also point at differences between positive and negative selection. Unlike in positively selected lineages, in negatively selected lineages, a smaller proportion of substituted sites are buried *β-Sheet* sites compared to all sites. This suggests the difference on positively selected lineages is not simply due to lower fragility in *β-Sheet* structure, but an active role for *β-Sheet* internal structure in driving evolution of new functionality. It should also be considered that, in general, positively selected lineages have fewer *α-Helix* (30.1108% vs 32.7617%) and more *β-Sheet* (21.7820% vs 19.8385%) sites compared to negatively selected lineages. Since, as discussed earlier, certain gene families and pathways are under more frequent positive selection than others, the lower selective constraint on *β-Sheet* sites has a long term impact on protein structure.

*β-Bridge* sites did not show a reduction in prevalence for substitutions on positively selected lineages. As these sites are used to hydrogen bond, particularly between *β-sheets*, the most likely source for these substitutions is to allow for protein restructuring. Purely compensatory driven changes are a less likely explanation, as negatively selected lineages where they are more likely than positively selected ones show a reduction in *β-Bridge* prevalence amongst substituted sites.

It should be noted that the same PDB structure is assumed to be applicable to all sequences in a gene family. As sequence pairs with divergence > 20% were split into separate families and as the median pairwise comparison among family members was 85% identity, the slow divergence of structural RMSD makes this a reasonable approximation [36]. Over longer evolutionary times [68, 69] and especially after lateral transfer events [60], repeated regions are known to lead to structural divergence.

### Gene family analysis of ornithine decarboxylase

Lastly TAED can be a valuable resource in understanding the lineage-specific evolution of individual gene families. To examine this, one gene family was selected based on criteria that it contained KEGG pathway mappings and structural information. The gene family that was analyzed encoded a putative ornithine decarboxylase. Ornithine decarboxylase is responsible for the decarboxylation of L-ornithine to putrescine. L-ornithine is a key component to the urea cycle and the decarboxylation of L-ornithine signals the irreversible reaction of forming putrescine which is the first step in polyamine synthesis [59]. Polyamines are polycations able to bind negatively charged molecules such as DNA and RNA. Three primary polyamines are important regulators of the MAPK pathway which plays a role in cell proliferation: putrescine, spermidine, and spermine. Spermidine is produced from putrescine which can further impact apoptosis [50]. As these molecules play an important role in cell growth and cellular death, the committed step in the synthesis of polyamines would be hypothesized to be evolving under strong negative constraint.

**Fig. 2:**
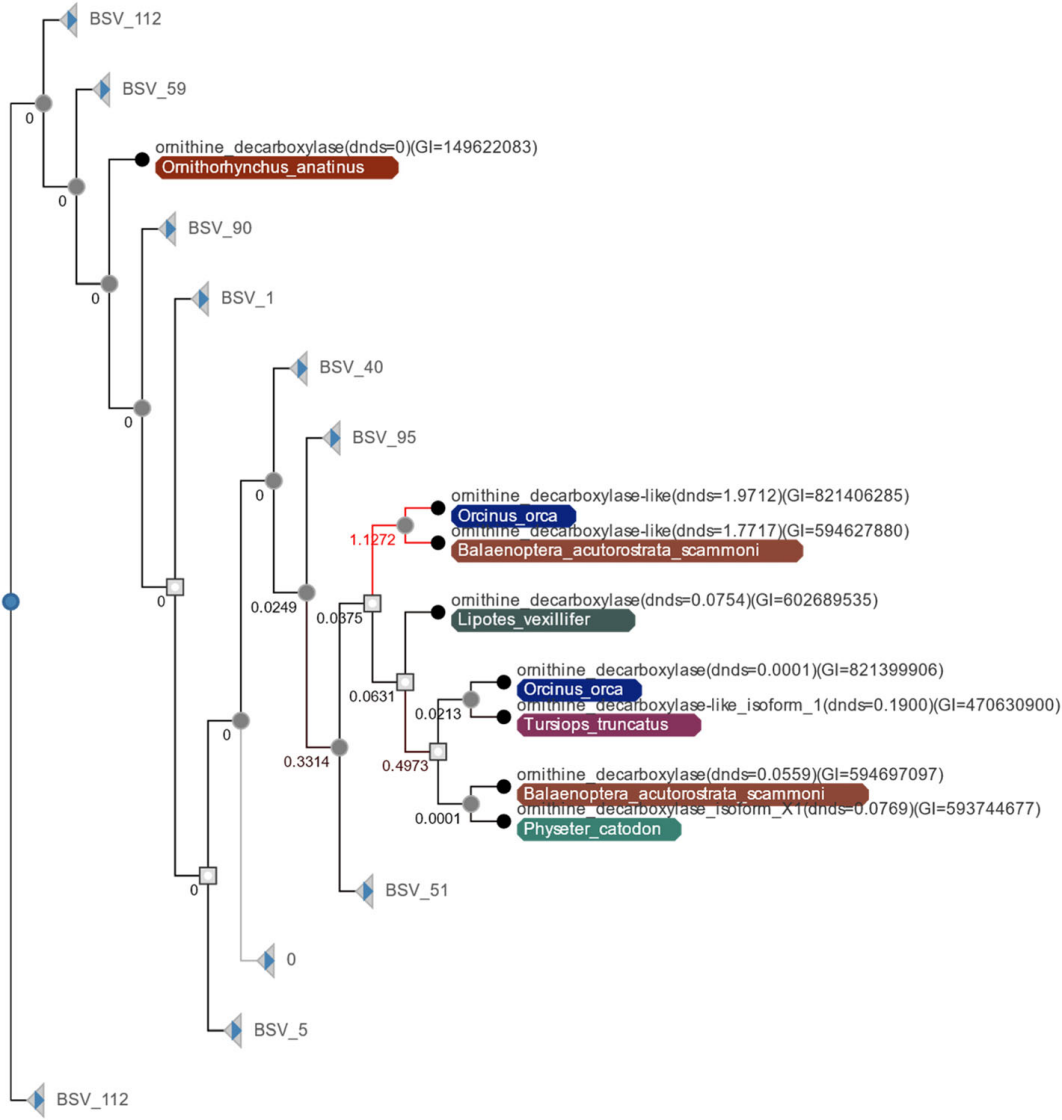
Gene tree for cetacean lineages of ornithine decarboxylase – Presented here is the gene tree taken from the TAED Tree Viewer for the TAED gene family 557. Lineages not associated with Cetaceans are collapsed. Internal nodes labeled with a while box are duplication events found within the tree. Nodes with solid grey dots represent speciation events. Nodes labeled in black indicate a leaf node. Lineages labeled in red have a dN/dS > 1 and the numbers along each branch are the associated dN/dS value for the given branch. Image was generated from the TAED Tree Viewer

An analysis of the TAED gene family showed six lineages with dN/dS > 1. These rates varied from a dN/dS rate of 2.0096 to 1.5451 (Table 9). Directional selection was found to have occurred on the lineage leading to Afrotherian mammals which are primarily localized to the continent of Africa and include: moles, elephants, manatees, and aardvarks. Other lineages with elevated rates of evolution were found for both *Macaca mulatta* (Rhesus macaque) and *Dasypus novemcinctus* (Nine-banded armadillo). Lastly, three different lineages involved cetacean species which may reflect the evolutionary pressures of moving from a terrestrial to an aquatic lifestyle. It was found that these instances of positive selection occurred following a duplication event, suggesting that the ornithine decarboxylase duplicate gene may have been under relaxed selective constraint following the duplication and not under the same strong constraints imposed by the polyamine synthesis pathway (Fig. 2). Although, since this protein was maintained and not lost over the 34 MYA of divergence between *Orcinus orca* (Killer whale) and *Balaenoptera acutorostrata scammoni* (Minke whale), it is likely that it has retained some functionality within these organisms.

**Table 9:** Lineages with dN/dS > 1 in Ornithine decarboxylase family

Lineages with dN/dS > 1 in TAED family for Ornithine decarboxylase			
dN/dS Value	Branch Start	Branch End	Mapped Branches
2.0096	Eutheria	Afrotheria	Afrotheria
1.9244	*Dasypus novemcinctus*	*Dasypus novemcinctus*	*Dasypus novemcinctus*
1.9712	Cetacea	*Orcinus orca*	*Ocinus orca*, Orcinus, Delphinidae, Odontoceti
1.7717	Cetacea	*Balaenopter acutorostrata scammoni*	*Balaenoptera acutorostrata scammoni*, *Balaenoptera acutorostrata*, Balaenoptera, Balaenopteridae, Mysticeti
1.1272	Cetacea	Cetacea	Cetacea
1.5451	Macaca	*Macaca mulatta*	*Macaca mulatta*

**Fig. 3:**
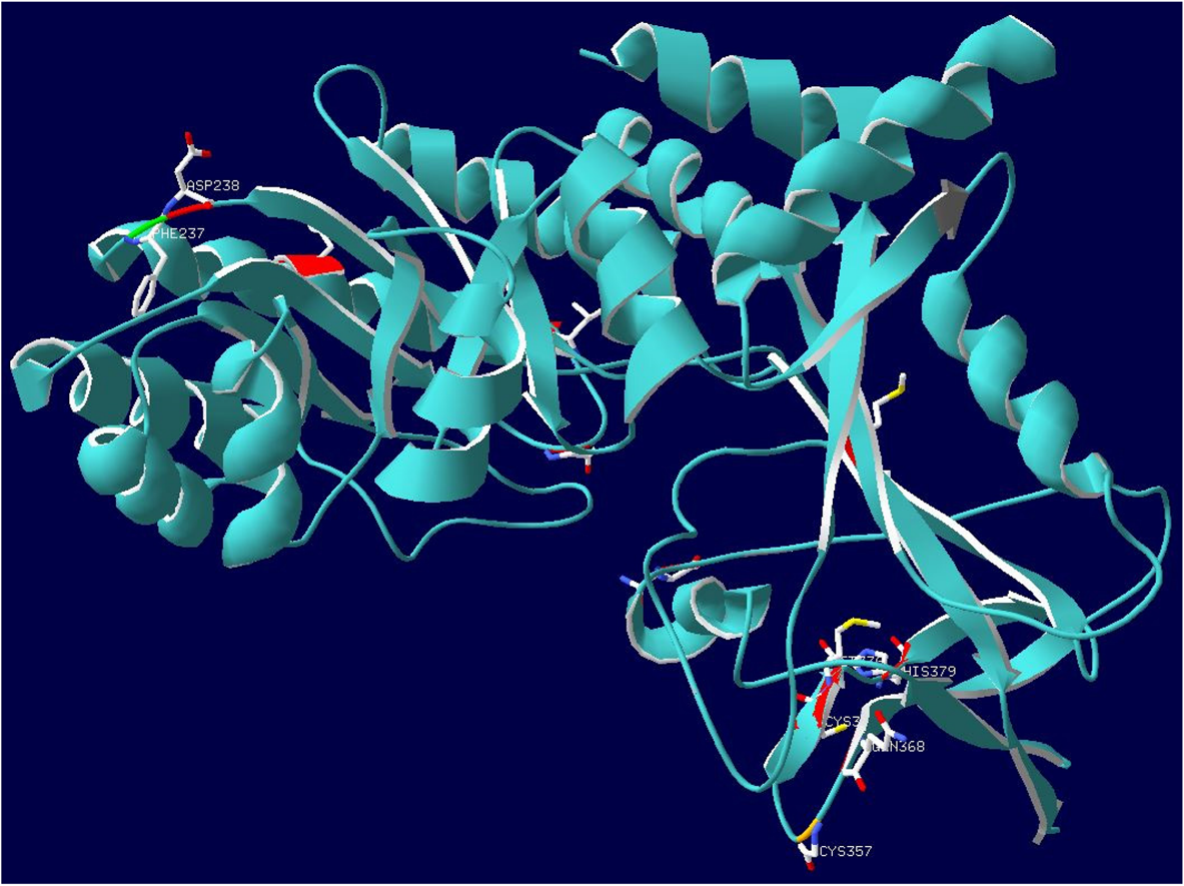
Pyridoxal phosphate binding site for ornithine decarboxylase along the lineage of Cetacea – A protein homology model of the ancestral protein leading to Cetacea was created. Template for the model was from human ornithine decarboxylase (PDB:2OO0; chain A). Ancestral changes occurring on the lineage for Cetacea have been mapped to the model, sites colored in red indicate nonsynonymous changes in the ancestral protein, sites colored in dark grey are synonymous site changes. The site indicated in green is the pyridoxal phosphate binding site 238. The site adjacent to the binding site is the substitution N238D found on the ancestral lineage. Image was generated from Swiss-PdbViewer

To better understand the molecular mechanisms associated with the increased rate of evolution detected within the evolution of ornithine decarboxylase in cetaceans an examination of the ancestral changes mapped to the extant version of human ornithine decarboxylase was performed. For the changes on the branch Cetacea, it was seen that a nonsynonymous substitution occurred at site 238 with an asparagine substituting to an aspartic acid (N238D). This substitution is situated one residue from site 237 which is a known pyridoxal phosphate binding site [22] (Fig. 3. The decarboxylation of L-ornithine to putrescine is known to be a pyridoxal 5′-phosphate dependent reaction [37] and therefore changes to this site in the protein may impact the rate or ability to catalyze L-ornithine. The N238D substitution caused a substitution for an uncharged amino acid to be replaced by a negatively charged amino acid which could potentially impact the pyridoxal phosphate binding site (Fig. 3).

**Fig. 4:**
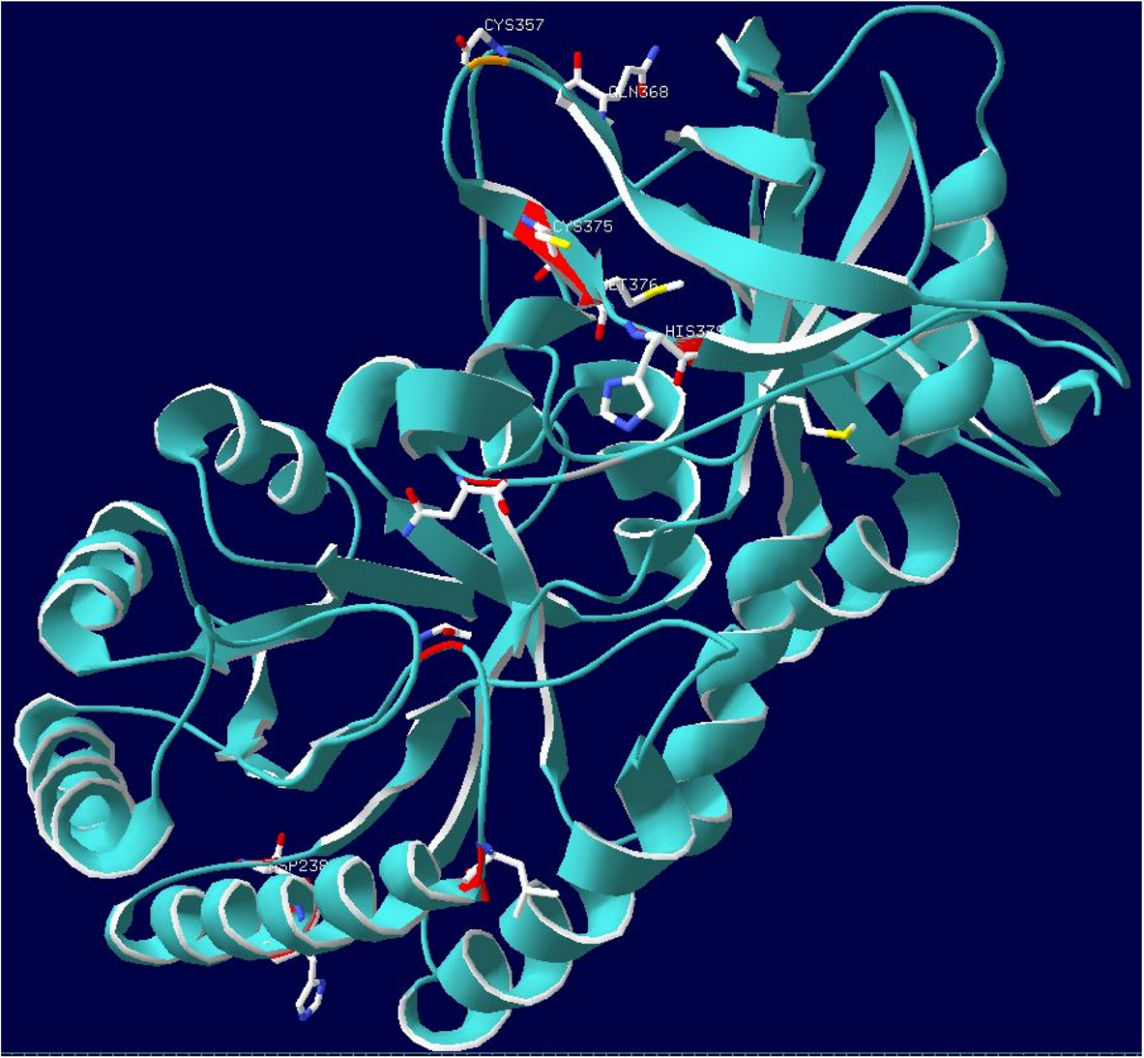
Active site remodeling for ornithine decarboxylase along the lineage of Cetacea – A protein homology model of the ancestral protein leading to Cetacea was created. Template for the model was from human ornithine decarboxylase (PDB:2OO0; chain A). Ancestral changes occurring on the lineage for Cetacea have been mapped to the model, sites colored in red indicate nonsynonymous changes in the ancestral protein, sites colored in dark grey are synonymous site changes. The site indicated in gold is the active site cysteine-357. Remodeling of the active site can be seen in the changes P368Q, R375C, I376M, and R379H which are positioned around the loop containing the active site

The active site of ornithine decarboxylase in humans is at residue 357 (Cystine - 357) [3]. While no substitutions were found at the active site, four different nonsynonymous substitutions were localized on the beta-sheets surrounding the active site. The substitutions P368Q, R375C, I376M, and R379H were all proximally close to the active site and may have been involved in remodeling of the active site for the cetacean duplicate of ornithine decarboxylase (Fig. 4). These mutations have impacted the ability of the protein in several ways, by either helping to stabilize the active site, change the specificity of the binding pocket, change the rate of the reaction, or cause the active site to become inert. Further experimental validation would be necessary to understand how the N238D substitution and the putative remodeling of the active site may impact the function of the protein. However, evidence from TAED does suggest that cetacean ornithine decarboxylase has undergone functional shifts in several different sites which may impact the efficacy of the decarboxylation of L-ornithine to putrescine. Why this enzyme would be under selection within Cetaceans is also an unanswered question, but understanding the lineage-specific evolution of ornithine decarboxylase may help to decipher the mechanistic reasons for how cetaceans were able to readapt to life in the water.

## Discussion

Understanding the mechanistic reasons that species diverge is of central importance to the field of molecular evolution. Gaining insight into how individual proteins evolve in context of the pathways in which they occur may help elucidate the underlying molecular mechanisms of speciation. Placing evolutionary events in the context of a species tree allows the interpretation of understanding how selective forces have varied across species. Here we have presented findings from The Adaptive Evolution Database (TAED) that have attempted to characterize the lineage-specific evolution of chordates. We know that selection can act on multiple levels within an organism, from the level of individual nucleotides to phenotypic traits in a population. We therefore have examined the effects of directional selection at the domain level, gene level, and pathway level to better understand the dynamics of lineage-specific evolution. Examination of high level trends within TAED have confirmed that some pathways including those that are related to metabolism, immunity, and cell signaling have been repeated targets for functional change and may play important roles in species divergence. Additionally, we have shown that some protein families have undergone many duplication events which have impacted the evolutionary constraints of the duplicate pairs. These duplicated genes may evolve to new functions within the genome and develop new links within pathways. Tools developed on TAED can be utilized to find gene families that have undergone instances of adaptive evolution and help to propose hypotheses for how these genes have evolved.

Not all parts of a protein are under the same selective constraints and residues located on the outside or surface of a protein may be more likely to evolve, and evolve at a different rate, than a residue which comprises the hydrophobic core of the protein. Our comparison of the solvent accessible surface area (SASA) and dN/dS showed that this holds for both positively selected and negatively selected lineages. It distinguishes differences between the action of the two kinds of selection beyond this by showing that while solvent accessibility is more exclusively the primary driver of changes in the nature of substituted sites on negatively selected lineages, positively selected lineages show relaxed selective constraint on β-Sheet and strengthen constraints on α-Helix sites.

Additionally, the relationship between the energetics of different substitutions and how they interplay with dN/dS could be explored by comparing dN/dS to the change in the change of free energy (ΔΔG) of a protein when different substitutions are introduced. Studies of this nature have examined how the thermodynamics of a protein influence the rate of dN/dS and how compensatory substitutions affect protein stability [61, 70]. Current evolutionary tests do not consider epistatic relationships within proteins, treating each site as acting independently from a statistical perspective.

Further, it is known that when N_e_ is large, selection is more efficient and the chance of an allele being lost from the population is small. However, when N_e_ is small the effects of genetic drift are greater and selection is less efficient [49, 75]. As such selection has limited ability to eliminate deleterious variants in chordates or fix advantageous changes, as chordate species have low effective populations sizes. Weber, et al. [80] found an unexpected negative correlation between N_e_ and dN/dS in bird populations, but found expected signals when considering the magnitude of biophysical effects of changes [80, 81].

TAED as a tool and resource in detecting episodes of lineage-specific evolution may also be useful in helping to understand the differences between directional selection and intra- and inter- molecular forces. Not all amino acid substitutions are the direct result of directional selection acting on a protein to functionally evolve. When physical changes within a molecule do occur, corresponding compensatory changes can occur which alleviate the deleterious effects of a mutation. These compensatory changes ensure that the newly substituted amino acid becomes the preferred amino acid for the residue in which it is located [61, 70]. Using traditional approaches of dN/dS it is difficult to differentiate between directional selection and compensatory changes as both aggregate across the branch. However, by examining changes in a lineage-specific context and determining when each substitution occurred along the lineage, it may be possible to begin to differentiate between these two processes.

The secondary structure analysis raises questions about the nature of the selective pressures on a protein-structure level, and points to the need for further investigation of β-sheet, α-helix, and 3_10_ Helix structures and their role in protein evolution in particular.

## Conclusions

TAED is a useful tool for understanding lineage-specific evolution and provides a source of data to develop further hypothesis-based inquiries into the mechanisms that drive diversification. In addition to providing an example of lineage-specific evolution in cetaceans, this work examined gene family evolution through the lenses of protein structure, co-evolution in pathways, as well as characterizing the duplication process within families. At the structural level, the study utilized the database to understand the differential patterns of amino acid substitution, including filtering by secondary structure, in comparing proteins under negative and positive selection. Overall, this work provides a further empirical window into the lineage-specific processes of evolution.

## Methods

### Database construction

The TAED database was constructed following the pipeline outlined in Hermansen et al. [33]. The pipeline includes generation of gene families from single-linkage clustering of BLAST results from chordate genes found on GenBank. A point accepted mutation (PAM) distance threshold of 120 was used for gene family construction. Gene families were refined for quality using an iterative method controlling for pairwise percent identity (> 80%) and the fraction of pairwise aligned gaps (< 10%). Gene families where then aligned using MAFFT [41] and phylogenetic trees were constructed using PhyML [30]. Gene tree – species tree reconciliation against the NCBI chordate taxonomy was implemented to determine putative duplication events and gene tree roots using SoftParsMap. Gene families were defined phylogenetically by the species tree except in cases where alignment quality prohibited this, as described here and in Hermansen et al. [33] (see [4] for a recent discussion of gene family construction methodology). Putative rates of evolution were then calculated using the branches model from PAML and dN/dS rates was computed. BLAST was then performed on TAED gene families against the KEGG database [40] to determine KEGG pathway relatedness and against PDB [10] to determine protein structure for each gene in TAED. All branches, including specifically those found to have a dN/dS > 1 (putatively evolving under positive selection) were mapped to the corresponding chordate species tree to determine along what lineage the elevated rates of evolution occurred and which proteins evolved rapidly on the same species tree lineage. Roots of all genes families were additionally mapped to the chordates species tree. To determine the approximate family root age for each gene family, information from TimeTree [43] was collected and root ages determined in MYA (millions of years ago). Domain classification information was gathered from the CATH database [71]. Putative functional annotations were assigned to each gene family based on NCBI nomenclature and KEGG pathway annotations when available.

### Enrichment analysis

Over/under-represented KEGG pathway and domain analyses were performed with a BLAST search against the KEGG database of TAED gene families. KO numbers were assigned to each individual protein in TAED that contained a BLAST hit with an e-value <1e^− 10^. This threshold was set so that all putative hits would be the result of orthologous descent instead of chance. The KO number from the top BLAST result was assigned to each TAED gene. KO numbers were then used to assess each putative biological pathway in which the protein is known to play a role. Over/under-representation of these pathways was then calculated using Fisher’s Exact test [23] and significance was estimated using an α-level of 0.05. The resulting *p*-values were corrected for multiple testing by performing a false discovery rate (FDR) analysis [8] with an FDR threshold of 0.05 and using a Bonferroni correction [13]. The FDR calculation was computed using the R statistical programming package [62]. A similar method was used to determine the over/under-representation of CATH domain topologies. The topology level classification was used as it represented a broad enough group that multiple topologies were found throughout TAED.

### Duplication analysis

For each gene family in TAED, the root node of the family was mapped to its associated lineage on the chordate species tree. Nodes were then given approximate dates in MYA based on estimates from the TimeTree database [43]. The number of duplication events that occurred in each gene family was used as inferred by SoftParsMap [9] through reconciliation with the NCBI taxonomy for chordates. A linear regression was performed on the resulting comparison between the family root node ages and the number of duplication found within each gene family. The Pearson’s r coefficient was calculated for the resulting linear regression with a Pearson’s r = 0.59. Log scaled transformations of the data did not yield a strong regression coefficient.

Since families were sought that showed a high propensity for duplicability in a short amount of time, families that fell below the regression line were filtered out (Fig. 1). We also filtered out all families whose length was below the 5th percentile, since evolutionary forces may not have had time to act on families with so few substitutions. Outliers in the resulting set of families were detected using Cook’s distance [20], which measures the change in regression coefficients due to the removal of a data point, and is often used as a proxy for the influence of that point. Gene families were then sorted according to this distance (Table 6). Finally, the top quartile of families was measured using this distance and the number of times they occur in each KEGG pathway was counted (Table 7).

Additionally, to test how different proxies of time impacted the duplication analysis, two additional proxies for time were generated: the maximum tree length, and the median tree length. The maximum tree length estimated in substitutions per site was calculated for all gene tree topologies by taking the maximal tree length from root to leaf node for every TAED gene family as estimated by PhyML. The median tree length was calculated in a similar manner by taking the median of all distances between the root and leaf of the phylogenetic tree for each gene family. Additional file 1: Figures S1 and S2 illustrate the differences in the duplication distribution of the families based on the change of the time component to the analysis. Each axis was of the analysis was given the transformation y = log (1 + x) and the Pearson’s r coefficient was calculated. The resulting best coefficients for both the maximum tree length and the median tree were found when both axes were log-transformed. Cook’s distance was calculated for each proxy of time and the families with pathways from the families with the largest Cook’s distance to the regression line were tabulated.

### Protein structure based analysis

Protein information was determined from stored PDB information associated with each gene family. To show that sites in different locations and belonging to different structures evolve at different rates, DSSP [39] values were used to ascertain the relative solvent accessibility (RSA) and secondary structure of individual sites within the protein was obtained. While newer and less approximate, but more computationally intensive methods than DSSP are available, a pilot analysis suggested that DSSP and more computationally intensive methods gave similar results for the purposes of this study. Membrane proteins and multimers were removed from the dataset based on identifying information in the PDB data. Sites were binned based on RSA using maximum surface areas from Tien et al. [77]; sites with a ratio greater than 0.20 were marked as exposed and buried otherwise, and then further categorized according to secondary structure. PAML analysis was used to determine the maximum likelihood ancestral sequence for each gene associated with a protein and the results controlled for lineages with dN/dS > 1 and lineages with a dN/dS <  0.5. dN/dS values of 0 or between 0.5 and 1 were ignored, as were any sites that did not align with the PDB sequence or were not one of the most common 20 amino acids. To determine the significance of the calculated values, two-tailed non-parametric bootstrapping was performed. For each lineage, simulated datasets of size matching the total substituted residue count were generated, using the distribution of all sites on the respective lineages as a baseline.

### Gene family analysis of ornithine decarboxylase

To demonstrate the application of lineage-specific analyses of evolution on specific gene families using TAED data, a gene family was selected for analysis based on the criteria that the gene family contained 3 or more lineages with dN/dS > 1 and it contained lineages that mapped to KEGG pathways and to a PDB structure. Using these criteria, the TAED gene family 554 (ornithine decarboxylase) was selected for further examination of lineage-specific evolution. dN/dS estimates of each lineage were taken from the TAED database. A homology model was generated using Swiss-Model [11], with the automated build method. The top template used in the homology model was PDB entry 2OO0 chain A. Ancestral amino acids were mapped to the model. Active site and binding site information was taken from the PDB website for the same entry. Uniprot [7] data for ornithine decarboxylase was also used to make inferences into important catalytic sites within the molecule. Images of the homology model were generated using Swiss-PdbViewer [29].

## Supplementary information

**Additional file 1: Figure S1.** The logarithm of the maximum phylogenetic tree length of TAED gene families, regressed against the logarithm of the number of duplications in a given family. Maximum length consists of the maximum cumulative branch length from the corresponding phylogenetic trees, considering all paths from root to tips. A log-scale was chosen since it resulted in a higher correlation coefficient. **Figure S2.** The logarithm of the median phylogenetic tree length of TAED gene families, regressed against the logarithm of the number of duplications in a given family. Meidan length consists of the median cumulative branch length from the corresponding phylogenetic trees, considering all paths from root to tips. A log-scale was chosen since it resulted in a higher correlation coefficient. **Table S1.** TAED gene families with many duplications based on maximum tree length. **Table S2.** TAED gene families with many duplications based on median tree length. **Table S3.** KEGG Pathways with many duplications based on maximum tree length. **Table S4.** KEGG Pathways with many duplications based on median tree length.

## Data Availability

The TAED Database is accessible at https://liberles.cst.temple.edu/TAED/index.html. Code available upon request.

## References

[1] Aik W, McDonough MA, Thalhammer A, Chowdhury R, Schofield CJ (2012). Role of the jelly-roll fold in substrate binding by 2-oxoglutarate oxygenases. Curr Opin Struct Biol.

[2] Aken BL, Achuthan P, Akanni W, Amode MR, Bernsdorff F, Bhai J (2017). Ensembl 2017. Nucleic Acids Res.

[3] Almrud JJ, Oliveira MA, Kern AD, Grishin NV, Phillips MA, Hackert ML (2000). Crystal structure of human ornithine decarboxylase at 2.1 å resolution: structural insights to antizyme binding. J Mol Biol.

[4] Altenhoff AM, Boeckmann B, Capella-Gutierrez S, Dalquen DA, DeLuca T, Forslund K, Huerta-Cepas J, Linard B, Pereira C, Pryszcz LP, Schreiber F, da Silva AS, Szklarczyk D, Train CM, Bork P, Lecompte O, von Mering C, Xenarios I, Sjölander K, Jensen LJ, Martin MJ, Muffato M, Gabaldón T, Lewis SE, Thomas PD, Sonnhammer E, Dessimoz C, Quest for Orthologs consortium (2016). Standardized benchmarking in the quest for orthologs. Nat Methods.

[5] Anisimova M, Yang Z (2007). Multiple hypothesis testing to detect lineages under positive selection that affects only a few sites. Mol Biol Evol.

[6] Anisimova Maria, Liberles David A. (2012). Detecting and understanding natural selection. Codon Evolution.

[7] Apweiler R, Bairoch A, Wu CH, Barker WC, Boeckmann B, Ferro S (2004). UniProt: the universal protein knowledgebase. Nucleic Acids Res.

[8] Benjamini Yoav, Hochberg Yosef (1995). Controlling the False Discovery Rate: A Practical and Powerful Approach to Multiple Testing. Journal of the Royal Statistical Society: Series B (Methodological).

[9] Berglund-Sonnhammer A-C, Steffansson P, Betts MJ, Liberles DA (2006). Optimal gene trees from sequences and species trees using a soft interpretation of parsimony. J Mol Evol.

[10] Berman HM, Westbrook J, Feng Z, Gilliland G, Bhat TN, Weissig H (2000). The protein data bank. Nucleic Acids Res.

[11] Biasini M, Bienert S, Waterhouse A, Arnold K, Studer G, Schmidt T (2014). SWISS-MODEL: modelling protein tertiary and quaternary structure using evolutionary information. Nucleic Acids Res.

[12] Bloch NI, Morrow JM, Chang BSW, Price TD (2015). SWS2 visual pigment evolution as a test of historically contingent patterns of plumage color evolution in warblers. Evolution.

[13] Bonferroni C (1936). Teoria statistica delle classi e calcolo delle probabilita. Pubblicazioni Del R Istituto Superiore Di Scienze Economiche e Commericiali Di Firenze.

[14] Boucher JI, Bolon DNA, Tawfik DS (2016). Quantifying and understanding the fitness effects of protein mutations: laboratory versus nature. Protein Sci.

[15] Brockhurst MA, Chapman T, King KC, Mank JE, Paterson S, Hurst GDD (2014). Running with the red queen: the role of biotic conflicts in evolution. Proc R Soc B Biol Sci.

[16] Brown SD, Babbitt PC (2014). New insights about enzyme evolution from large scale studies of sequence and structure relationships. J Biol Chem.

[17] Cheng S, Brooks CL (2013). Viral capsid proteins are segregated in structural fold space. PLoS Comput Biol.

[18] Chi PB, Kim D, Lai JK, Bykova N, Weber CC, Kubelka J, Liberles DA (2018). A new parameter-rich structure-aware mechanistic model for amino acid substitution during evolution. Proteins.

[19] Chi PB, Liberles DA (2016). Selection on protein structure, interaction, and sequence. Protein Sci.

[20] Cook RD (1977). Detection of influential observation in linear regression. Technometrics.

[21] Deming Paula B., Campbell Shirley L., Stone Jamie B., Rivard Robert L., Mercier Alison L., Howe Alan K. (2015). Anchoring of Protein Kinase A by ERM (Ezrin-Radixin-Moesin) Proteins Is Required for Proper Netrin Signaling through DCC (Deleted in Colorectal Cancer). Journal of Biological Chemistry.

[22] Dufe VT, Ingner D, Heby O, Khomutov AR, Persson L, Al-Karadaghi S (2007). A structural insight into the inhibition of human and Leishmania donovani ornithine decarboxylases by 1-amino-oxy-3-aminopropane. Biochem J.

[23] Fisher RA (1922). On the interpretation of χ 2 from contingency tables, and the calculation of P. J R Stat Soc.

[24] Gatesy J, Geisler JH, Chang J, Buell C, Berta A, Meredith RW (2013). A phylogenetic blueprint for a modern whale. Mol Phylogenet Evol.

[25] Gharib WH, Robinson-Rechavi M (2013). The branch-site test of positive selection is surprisingly robust but lacks power under synonymous substitution saturation and variation in GC. Mol Biol Evol.

[26] Glusman G, Bahar A, Sharon D, Pilpel Y, White J, Lancet D (2000). The olfactory receptor gene superfamily: data mining, classification, and nomenclature. Mamm Genome.

[27] Goldman AD, Beatty JT, Landweber LF (2016). The TIM barrel architecture facilitated the early evolution of protein-mediated metabolism. J Mol Evol.

[28] Grahnen JA, Nandakumar P, Kubelka J, Liberles DA (2011). Biophysical and structural considerations for protein sequence evolution. BMC Evol Biol.

[29] Guex N, Peitsch MC, Schwede T (2009). Automated comparative protein structure modeling with SWISS-MODEL and Swiss-PdbViewer: a historical perspective. Electrophoresis.

[30] Guindon S, Dufayard J-F, Lefort V, Anisimova M, Hordijk W, Gascuel O (2010). New algorithms and methods to estimate maximum-likelihood phylogenies: assessing the performance of PhyML 3.0. Syst Biol.

[31] Hanukoglu I (2015). Proteopedia: Rossmann fold: a beta-alpha-beta fold at dinucleotide binding sites. Biochem Mol Biol Educ.

[32] Hermansen RA, Mannakee BK, Knecht W, Liberles DA, Gutenkunst RN (2015). Characterizing selective pressures on the pathway for de novo biosynthesis of pyrimidines in yeast. BMC Evol Biol.

[33] Hermansen RA, Oswald BP, Knight S, Shank SD, Northover D, Korunes KL (2017). The adaptive evolution database (TAED): a new release of a database of Phylogenetically indexed gene families from chordates. J Mol Evol.

[34] Horowitz NH (1945). On the evolution of biochemical syntheses. Proc Natl Acad Sci U S A.

[35] Hughes T, Liberles DA (2007). The pattern of evolution of smaller-scale gene duplicates in mammalian genomes is more consistent with neo- than subfunctionalisation. J Mol Evol.

[36] Illergård K, Ardell DH, Elofsson A (2009). Structure is three to ten times more conserved than sequence--a study of structural response in protein cores. Proteins.

[37] Jackson LK, Baldwin J, Akella R, Goldsmith EJ, Phillips MA (2004). Multiple active site conformations revealed by distant site mutation in ornithine decarboxylase. Biochemistry.

[38] Jensen RA (1976). Enzyme recruitment in evolution of new function. Annu Rev Microbiol.

[39] Kabsch W, Sander C (1983). Dictionary of protein secondary structure: pattern recognition of hydrogen-bonded and geometrical features. Biopolymers.

[40] Kanehisa M, Sato Y, Kawashima M, Furumichi M, Tanabe M (2016). KEGG as a reference resource for gene and protein annotation. Nucleic Acids Res.

[41] Katoh K, Standley DM (2013). MAFFT multiple sequence alignment software version 7: improvements in performance and usability. Mol Biol Evol.

[42] Konrad A, Teufel AI, Grahnen JA, Liberles DA (2011). Toward a general model for the evolutionary dynamics of gene duplicates. Genome Biol Evol.

[43] Kumar S, Stecher G, Suleski M, Hedges SB (2017). TimeTree: a resource for timelines, Timetrees, and divergence times. Mol Biol Evol.

[44] Kurtz J, Schulenburg H, Reusch TBH (2016). Host–parasite coevolution—rapid reciprocal adaptation and its genetic basis. Zoology.

[45] Laurino P, Tóth-Petróczy Á, Meana-Pañeda R, Lin W, Truhlar DG, Tawfik DS (2016). An ancient fingerprint indicates the common ancestry of Rossmann-fold enzymes utilizing different ribose-based cofactors. PLoS Biol.

[46] Liberles DA, Schreiber DR, Govindarajan S, Chamberlin SG, Benner SA (2001). The adaptive evolution database (TAED). Genome Biol.

[47] Liberles DA, Tisdell MDM, Grahnen JA (2011). Binding constraints on the evolution of enzymes and signalling proteins: the important role of negative pleiotropy. Proc Biol Sci.

[48] Light S, Kraulis P (2004). Network analysis of metabolic enzyme evolution in Escherichia coli. BMC Bioinformatics.

[49] Lynch M, Bobay L-M, Catania F, Gout J-F, Rho M (2011). The repatterning of eukaryotic genomes by random genetic drift. Annu Rev Genomics Hum Genet.

[50] Minois N, Carmona-Gutierrez D, Madeo F (2011). Polyamines in aging and disease. Aging.

[51] Moretti S, Laurenczy B, Gharib WH, Castella B, Kuzniar A, Schabauer H (2014). Selectome update: quality control and computational improvements to a database of positive selection. Nucleic Acids Res.

[52] Newfeld SJ, Wisotzkey RG, Kumar S (1999). Molecular evolution of a developmental pathway: phylogenetic analyses of transforming growth factor-beta family ligands, receptors and Smad signal transducers. Genetics.

[53] Newfeld SJ, Wisotzkey RG (2006). Molecular evolution of Smad proteins. Smad signal transduction.

[54] Niimura Y, Nei M (2007). Extensive gains and losses of olfactory receptor genes in mammalian evolution. PLoS One.

[55] Ohno S. Evolution by Gene Duplication. Berlin: Springer-Verlag; 1970.

[56] Orlenko A, Chi PB, Liberles DA (2017). Characterizing the roles of changing population size and selection on the evolution of flux control in metabolic pathways. BMC Evol Biol.

[57] Orlenko A, Teufel AI, Chi PB, Liberles DA (2016). Selection on metabolic pathway function in the presence of mutation-selection-drift balance leads to rate-limiting steps that are not evolutionarily stable. Biol Direct.

[58] Pang K, Cheng C, Xuan Z, Sheng H, Ma X (2010). Understanding protein evolutionary rate by integrating gene co-expression with protein interactions. BMC Syst Biol.

[59] Pegg AE (2006). Regulation of ornithine decarboxylase. J Biol Chem.

[60] Persi E, Wolf YI, Koonin EV (2016). Positive and strongly relaxed purifying selection drive the evolution of repeats in proteins. Nat Commun.

[61] Pollock DD, Thiltgen G, Goldstein RA (2012). Amino acid coevolution induces an evolutionary stokes shift. Proc Natl Acad Sci U S A.

[62] R Core Team. R: A Language and Environment for Statistical Computing: R Foundation for Statistical Computing; 2016.

[63] Rao ST, Rossmann MG (1973). Comparison of super-secondary structures in proteins. J Mol Biol.

[64] Raoult D, Forterre P (2008). Redefining viruses: lessons from Mimivirus. Nat Rev Microbiol.

[65] Rastogi S, Liberles DA (2005). Subfunctionalization of duplicated genes as a transition state to neofunctionalization. BMC Evol Biol.

[66] Roth C, Betts MJ, Steffansson P, Saelensminde G, Liberles DA (2005). The Adaptive Evolution Database (TAED): a phylogeny based tool for comparative genomics. Nucleic Acids Res.

[67] Sayers EW, Cavanaugh M, Clark K, Ostell J, Pruitt KD, Karsch-Mizrachi I. GenBank. Nucleic Acids Res. 2020;48(D1):D84–D86. 10.1093/nar/gkz956. 10.1093/nar/gkz956 PMC7145611 31665464

[68] Schaeffer RD, Kinch LN, Liao Y, Grishin NV (2016). Classification of proteins with shared motifs and internal repeats in the ECOD database. Protein Sci.

[69] Schüler A, Bornberg-Bauer E (2016). Evolution of protein domain repeats in Metazoa. Mol Biol Evol.

[70] Shah P, McCandlish DM, Plotkin JB (2015). Contingency and entrenchment in protein evolution under purifying selection. Proc Natl Acad Sci U S A.

[71] Sillitoe I, Lewis TE, Cuff A, Das S, Ashford P, Dawson NL (2015). CATH: comprehensive structural and functional annotations for genome sequences. Nucleic Acids Res.

[72] Spady Tyrone C., Seehausen Ole, Loew Ellis R., Jordan Rebecca C., Kocher Thomas D., Carleton Karen L. (2005). Adaptive Molecular Evolution in the Opsin Genes of Rapidly Speciating Cichlid Species. Molecular Biology and Evolution.

[73] Spielman SJ, Wilke CO (2015). The relationship between dN/dS and scaled selection coefficients. Mol Biol Evol.

[74] Sugawara T, Imai H, Nikaido M, Imamoto Y, Okada N (2010). Vertebrate rhodopsin adaptation to dim light via rapid meta-II intermediate formation. Mol Biol Evol.

[75] Sung W, Ackerman MS, Miller SF, Doak TG, Lynch M (2012). Drift-barrier hypothesis and mutation-rate evolution. Proc Natl Acad Sci U S A.

[76] Teufel AI, Liu L, Liberles DA (2016). Models for gene duplication when dosage balance works as a transition state to subsequent neo- or sub-functionalization. BMC Evol Biol.

[77] Tien MZ, Meyer AG, Sydykova DK, Spielman SJ, Wilke CO (2013). Maximum allowed solvent accessibilites of residues in proteins. PLoS One.

[78] Van Valen L (1974). Molecular evolution as predicted by natural selection. J Mol Evol.

[79] Weadick CJ, Loew ER, Rodd FH, Chang BSW (2012). Visual pigment molecular evolution in the Trinidadian pike cichlid (*Crenicichla frenata*): a less colorful world for neotropical cichlids?. Mol Biol Evol.

[80] Weber CC, Nabholz B, Romiguier J, Ellegren H (2014). K r /K c but not d N /d S correlates positively with body mass in birds, raising implications for inferring lineage-specific selection. Genome Biol.

[81] Weber Claudia C, Whelan Simon (2019). Physicochemical Amino Acid Properties Better Describe Substitution Rates in Large Populations. Molecular Biology and Evolution.

[82] Wierenga R (2001). The TIM-barrel fold: a versatile framework for efficient enzymes. FEBS Lett.

[83] Yang Z (1998). Likelihood ratio tests for detecting positive selection and application to primate lysozyme evolution. Mol Biol Evol.

[84] Zhang G, Li C, Li Q, Li B, Larkin DM, Lee C (2014). Comparative genomics reveals insights into avian genome evolution and adaptation. Science.

