# Supplementary material for: Characterizing lineage-specific evolution and the processes driving genomic diversification in chordates
Source: BMC Evol Biol. 2020 Feb 11;20:24. doi: 10.1186/s12862-020-1585-y (PMC7011509; doi:10.1186/s12862-020-1585-y)
Supplement: Supplementary file 1 — Additional file 1: Figure S1. The logarithm of the maximum phylogenetic tree length of TAED gene families, regressed against the logarithm of the number of duplications in a given family. Maximum length consists of the maximum cumulative branch length from the corresponding phylogenetic trees, considering all paths from root to tips. A log-scale was chosen since it resulted in a higher correlation coefficient. Figure S2. The logarithm of the median phylogenetic tree length of TAED gene families, regressed against the logarithm of the number of duplications in a given family. Meidan length consists of the median cumulative branch length from the corresponding phylogenetic trees, considering all paths from root to tips. A log-scale was chosen since it resulted in a higher correlation coefficient. Table S1. TAED gene families with many duplications based on maximum tree length. Table S2. TAED gene families with many duplications based on median tree length. Table S3. KEGG Pathways with many duplications based on maximum tree length. Table S4. KEGG Pathways with many duplications based on median tree length. [file 12862_2020_1585_MOESM1_ESM.docx]

| Northover et al., Supplemental Materials   \| **Figure S1 Legend:** \| \| --- \| |
| --- | --- |

The logarithm of the maximum phylogenetic tree length of TAED gene families, regressed against the logarithm of the number of duplications in a given family. Maximum length consists of the maximum cumulative branch length from the corresponding phylogenetic trees, considering all paths from root to tips. A log-scale was chosen since it resulted in a higher correlation coefficient.

| \| **Figure S2 Legend:** \| \| --- \| |
| --- | --- |

The logarithm of the median phylogenetic tree length of TAED gene families, regressed against the logarithm of the number of duplications in a given family. Meidan length consists of the median cumulative branch length from the corresponding phylogenetic trees, considering all paths from root to tips. A log-scale was chosen since it resulted in a higher correlation coefficient.

**Figure S1**

**
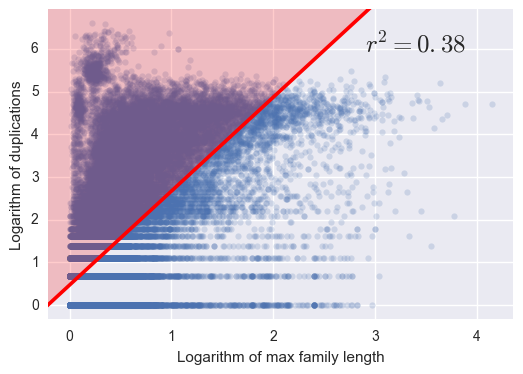
**

**Figure S2**

**
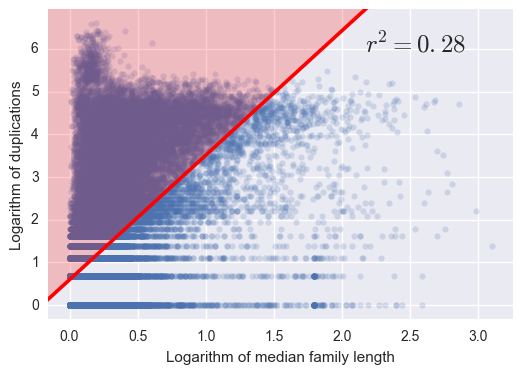
**

**Table S1****.** TAED gene families with many duplications based on maximum tree length.

| Cook’s Distance | Family Name | Family Length | Duplications |
| --- | --- | --- | --- |
| 0.000241 | abl interactor 1, partial | 0.05284 | 542 |
| 0.000228 | peptidyl-prolyl cis-trans isomerase A-like | 1.653113 | 586 |
| 0.000205 | protein SMG7 isoform X3 | 0.04828 | 332 |
| 0.000199 | rab GTPase-activating protein 1-like isoform X1 | 1.895474 | 404 |
| 0.000199 | RNA-binding protein Musashi homolog 2 | 1.888377 | 404 |
| 0.000194 | synaptosomal-associated protein 25 isoform X2 | 0.04862 | 288 |
| 0.000193 | mitogen-activated protein kinase kinase kinase kinase 4 isoform X4 | 0.124795 | 511 |
| 0.000183 | fragile X mental retardation syndrome-related protein 1 isoform X1 | 0.10182 | 370 |
| 0.000181 | protein transport protein Sec61 subunit alpha isoform 2 isoform X1 | 0.06523 | 277 |
| 0.000178 | amyloid beta A4 protein-like protein | 0.162232 | 525 |
| 0.000178 | far upstream element-binding protein 1 isoform X2 | 0.171275 | 554 |
| 0.000178 | myocyte enhancer factor 2c | 0.168375 | 542 |
| 0.000177 | dystrophin isoform X1 | 1.465036 | 429 |
| 0.000176 | nuclear transcription factor Y, gamma | 0.091775 | 315 |
| 0.000176 | AP-3 complex subunit sigma-2 | 2.272882 | 307 |
| 0.000174 | AN1-type zinc finger protein 6 isoform X1 | 0.06812 | 257 |
| 0.000171 | ubiquitin-conjugating enzyme E2 variant 2 | 0.1421 | 415 |
| 0.000169 | unnamed protein product | 0.18642 | 532 |
| 0.000168 | single-stranded DNA-binding protein 2 isoform X8 | 0.03617 | 185 |
| 0.000167 | serine/threonine-protein phosphatase 2A 56 kDa regulatory subunit epsilon isoform | 0.25149 | 729 |
| 0.000166 | myosin regulatory light polypeptide 9 | 0.17414 | 465 |
| 0.000164 | MAP/microtubule affinity-regulating kinase 3 | 0.05064 | 196 |
| 0.000164 | exocyst complex component 7 | 0.15776 | 410 |
| 0.000164 | rho GTPase-activating protein 12 isoform X4 | 0.10812 | 294 |

**Table S2.** TAED gene families with many duplications based on median tree length.

| Cook’s Distance | Family Name | Median Family Length | Duplications |
| --- | --- | --- | --- |
| 0.000183 | aldolase B | 0.829565 | 463 |
| 0.00018 | abl interactor 1, partial | 0.05281 | 542 |
| 0.000177 | myocyte enhancer factor 2c | 0.057375 | 542 |
| 0.000166 | mitogen-activated protein kinase kinase kinase kinase 4 isoform X4 | 0.067807 | 511 |
| 0.000164 | calmodulin isoform X2 | 0.02873 | 315 |
| 0.000163 | gephyrin | 0.02988 | 318 |
| 0.000161 | forkhead box P2 | 0.072697 | 497 |
| 0.000161 | ELAV-like protein 4 isoform X7 | 0.02554 | 289 |
| 0.000157 | amyloid beta A4 protein-like protein | 0.084298 | 525 |
| 0.000156 | abl interactor 2 | 0.085334 | 526 |
| 0.000153 | protein SMG7 isoform X3 | 0.04826 | 332 |
| 0.000151 | visinin-like protein 1 | 0.037 | 283 |
| 0.00015 | NMDA receptor synaptonuclear signaling and neuronal migration factor isoform X7 | 1.481947 | 222 |
| 0.000147 | LOW QUALITY PROTEIN: splicing factor U2AF 26 kDa subunit | 0.076776 | 408 |
| 0.000145 | ELAV-like protein 2, partial | 0.03166 | 240 |
| 0.000144 | synaptosomal-associated protein 25 isoform X2 | 0.04861 | 288 |
| 0.000144 | cellular nucleic acid-binding protein isoform X2 | 0.05864 | 319 |
| 0.000141 | LOW QUALITY PROTEIN: histone demethylase UTY-like | 0.104195 | 480 |
| 0.000141 | histone H2A.V isoform X1 | 0.057405 | 297 |
| 0.00014 | eyes absent-1 beta | 0.056439 | 293 |
| 0.00014 | ubiquitin-associated protein 2-like isoform X2 | 0.113547 | 519 |
| 0.00014 | nuclear transcription factor Y, gamma | 0.064265 | 315 |
| 0.000139 | DNA-directed RNA polymerases I, II, and III subunit RPABC3 | 1.330071 | 204 |
| 0.000139 | histone-lysine N-methyltransferase EZH2 isoform X2 | 0.084628 | 382 |

**Table S3.** KEGG Pathways with many duplications based on maximum tree length.

| Pathway | Duplication Count |
| --- | --- |
| Metabolic pathways | 981 |
| Olfactory transduction | 467 |
| Pathways in cancer | 219 |
| Neuroactive ligand-receptor interaction | 215 |
| PI3K-Akt signaling pathway | 203 |
| Endocytosis | 184 |
| Cytokine-cytokine receptor interaction | 178 |
| MAPK signaling pathway | 170 |
| HTLV-I infection | 161 |
| Focal adhesion | 159 |
| Regulation of actin cytoskeleton | 143 |
| Herpes simplex infection | 141 |
| Purine metabolism | 140 |
| Calcium signaling pathway | 125 |
| Proteoglycans in cancer | 124 |
| Rap1 signaling pathway | 123 |
| Ras signaling pathway | 120 |
| Cell adhesion molecules (CAMs) | 118 |
| RNA transport | 116 |
| Protein processing in endoplasmic reticulum | 116 |
| Huntington's disease | 115 |
| Phagosome | 114 |
| Epstein-Barr virus infection | 112 |
| cAMP signaling pathway | 111 |
| Influenza A | 110 |

**Table S4.** KEGG Pathways with many duplications based on median tree length.

| Pathway | Duplication Count |
| --- | --- |
| Metabolic pathways | 1027 |
| Olfactory transduction | 460 |
| Pathways in cancer | 236 |
| Neuroactive ligand-receptor interaction | 223 |
| PI3K-Akt signaling pathway | 214 |
| Endocytosis | 193 |
| Cytokine-cytokine receptor interaction | 183 |
| HTLV-I infection | 176 |
| MAPK signaling pathway | 175 |
| Focal adhesion | 160 |
| Regulation of actin cytoskeleton | 150 |
| Purine metabolism | 147 |
| Herpes simplex infection | 142 |
| Proteoglycans in cancer | 134 |
| Rap1 signaling pathway | 133 |
| Protein processing in endoplasmic reticulum | 133 |
| Calcium signaling pathway | 133 |
| Ras signaling pathway | 130 |
| Cell adhesion molecules (CAMs) | 127 |
| Epstein-Barr virus infection | 125 |
| RNA transport | 122 |
| Huntington's disease | 121 |
| Jak-STAT signaling pathway | 119 |
| Phagosome | 118 |
| cAMP signaling pathway | 117 |
